# Evaluation of Changes in Social Isolation and Loneliness with Incident Cardiovascular Events and Mortality

**DOI:** 10.1007/s44197-024-00243-3

**Published:** 2024-05-27

**Authors:** Yilin Chen, Huachen Xue, Yu Nie, Yujing Zhou, Sizhi Ai, Yaping Liu, Jihui Zhang, Yannis Yan Liang

**Affiliations:** 1 Center for Sleep and Circadian Medicine, The Affiliated Brain Hospital of Guangzhou Medical University, 36 Mingxin Road, 510370 Guangzhou, Guangdong China; 2 Department of Neurology and National Center for Neurological Disorders, State Key Laboratory of Medical Neurobiology and MOE Frontiers Center for Brain Science, Huashan Hospital, Fudan University, 200040 Shanghai, China; 3 Key Laboratory of Neurogenetics and Channelopathies of Guangdong Province and the Ministry of Education of China, Guangzhou Medical University, 510260 Guangzhou, Guangdong China; 4 Department of Cardiology, Heart Center, The First Affiliated Hospital of Xinxiang Medical University, 453199 Weihui, Henan China; 5 Institute of Psycho-neuroscience, The Affiliated Brain Hospital of Guangzhou Medical University, 510370 Guangzhou, Guangdong China; 6 Guangdong Cardiovascular Institute, Guangdong Provincial People’s Hospital, Southern Medical University, 510080 Guangzhou, Guangdong China

**Keywords:** Social isolation, Loneliness, Cardiovascular diseases, Mortality, UK Biobank

## Abstract

**Background:**

It remains unknown how the patterns of change of social isolation and loneliness are associated with the onset of cardiovascular disease (CVD) and mortality. We aimed to investigate the longitudinal association of changes in social isolation and loneliness with incident CVD, all-cause mortality, CVD mortality and subsequent cardiac function.

**Methods:**

This prospective cohort study included 18,258 participants aged 38–73 years who participated in visit 0 (2006–2010) and visit 1 (2012–2013) using UK Biobank (mean age 57.1, standard deviation [SD] 7.4; 48.7% males). Social isolation or loneliness was categorized into four patterns: never, transient, incident, and persistent. Incident CVD, all-cause and CVD mortality were ascertained through linkage data. Cardiac function was assessed by cardiovascular magnetic resonance imaging in a subsample (*N* = 5188; visit 2, since 2014).

**Results:**

Over a median follow-up of 8.3 (interquartile range [IQR] 8.1–8.6) years, compared with never social isolation, persistent social isolation was associated with the higher risk of incident CVD (hazard ratio [HR] 1.17, 95% confidence interval [CI] 1.03–1.33), all-cause (1.42, 1.12–1.81) and CVD (1.53, 1.05–2.23) mortality. Likewise, persistent loneliness was strongly associated with the greater risk of incident CVD (1.13, 1.00–1.27), all-cause (1.28, 1.02–1.61) and CVD mortality (1.52, 1.06–2.18).

**Conclusions:**

Persistent social isolation and loneliness posed a substantially higher risk for incident CVD, all-cause and CVD mortality, and cardiac dysfunction than other patterns. Persistent social isolation and loneliness, along with an increasing cumulative score, are associated with lower cardiac function.

**Graphical Abstract:**

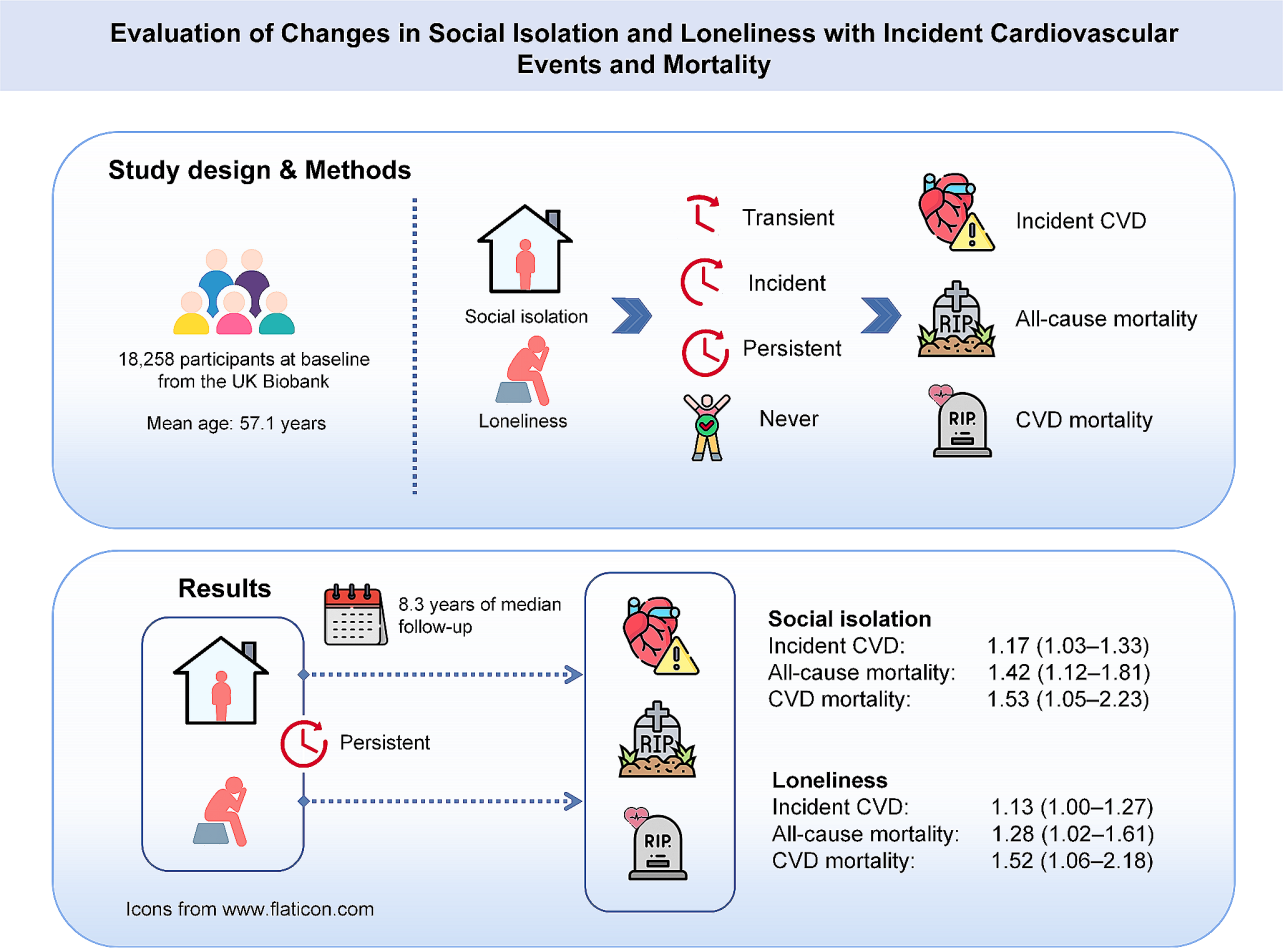

**Supplementary Information:**

The online version contains supplementary material available at 10.1007/s44197-024-00243-3.

## Introduction

Social disconnection, including social isolation and loneliness, has been recognized as modifiable risk factors for adverse health outcomes [1], particularly cardiovascular disease (CVD) and mortality [2–6]. Social isolation is defined as a lack of objective social contacts or being alone [1], while loneliness is a stressful experience where individuals subjectively perceive themselves to be socially isolated from others [7]. They are two weakly correlated but distinct constructs with dissimilar health effects [3]. The status of social isolation and loneliness may be dynamic and change over time due to newly occurring stressful life events or personal resilience to distressing events [8]. For instance, the rate of social isolation and loneliness has rapidly increased, especially since the beginning of the coronavirus disease 2019 (COVID-19) pandemic [1, 9].

Although there is abundant evidence on the association of social isolation and loneliness with cardiovascular health and survival [2–5], almost all previous studies applied a single assessment of social isolation or loneliness. Despite their prospective design, the observed association based on a single measurement failed to track the impact of change patterns or cumulative burden of social isolation and loneliness on health outcomes. Analyses that take into account the dynamics of social isolation and loneliness are needed to understand of the underlying mechanisms. Several recent studies have indicated that the negative impacts of loneliness on cognitive health vary according to its patterns of change [10, 11]. Similarly, Guo et al. have shown that the fluctuating and consistently high social isolation is associated with high risks of developing CVD. However, they only evaluated the association between changes of social isolation and onset of CVD but overlooked the mortality [12]. To our knowledge, how changes in social isolation and loneliness affect the risks of incident CVD and mortality remains largely unclear.

To fill this research gap, using follow-up data from the UK Biobank study, the present study aimed to investigate the longitudinal association between changes in social isolation and loneliness over a 4-year exposure period with incident CVD, all-cause and CVD mortality, and subsequent cardiac function measured using cardiovascular magnetic resonance (CMR) imaging.

## Materials and Methods

### Study Design and Population

**Fig. 1:**
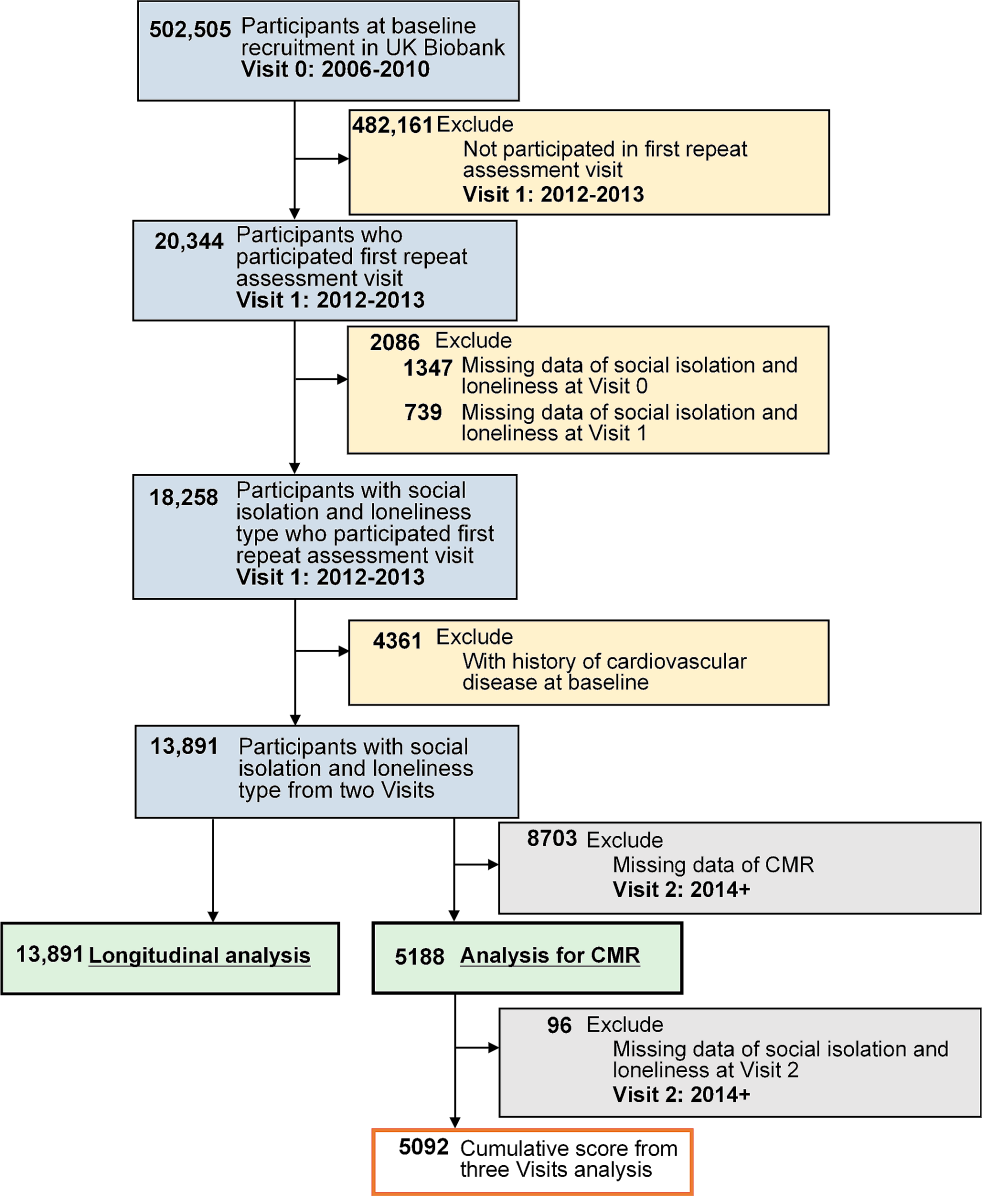
Flowchart of participant selection. CMR, cardiac magnetic resonance; CVD, cardiovascular disease

The UK Biobank is a prospective, population-based cohort study that enrolled 502,505 adults aged 38–73 years in the UK between 2006 and 2010 (visit 0) [13]. These participants were invited to attend one of 23 centers across England, Scotland, and Wales, where their information on lifestyle and health data, physical measurements, and biological samples were collected. The detailed description of the UK Biobank study design was provided elsewhere [14]. The present study was restricted to participants with complete data on social isolation and loneliness available at both visit 0 (2006–2010) and visit 1 (2012–2013). Overall, the final sample comprised 18,258 participants, and was used to assess the association between social isolation and loneliness and mortality. For analyses with incident CVD as the outcome, participants were excluded if they had a history of CVD before visit 1 (2012–2013). The participants underwent CMR imaging at visit 2 (imaging visit since 2014). A subsample of 5188 participants with available CMR data was included to test the associations of social isolation and loneliness with cardiac function. Details of the inclusion and exclusion criteria of the current study are displayed in Fig. 1.

### Exposures

#### Social Isolation

Participants were asked to answer questions regarding social isolation and loneliness at each visit. The social isolation index used by the UK Biobank, similar to the validated Berkman–Syme social network index [15], was constructed from the following three questions: (1) ‘How often do you visit friends or family or have them visit you?’ (1 point was given for answering about once a month, once every few months, never or almost never, or no friends or family outside the household); (2) “Which of the following (sports club or gym, pub or social club, religious group, adult education class, other group activity) do you engage in once a week or more often?” (1 point was given for answering none of the above); (3) “Including yourself, how many people are living together in your household?” (1 point was given for living alone). Using the scoring method implemented in previous studies [2, 6, 16], we constructed a social isolation index score ranged from 0 to 3, with a higher score indicating greater social isolation. The presence of social isolation was defined using a cut-off score of 2. Four patterns of change in social isolation were defined according to the status between visit 0 (2006–2010) and visit 1 (2012–2013): (1) never social isolation: absence of social isolation at both visits 0 and 1; (2) transient social isolation: being socially isolated only at visit 0 but not visit 1; (3) incident social isolation: being socially isolated only at visit 1 but not visit 0; and (4) persistent social isolation: presence of social isolation at both visits 0 and 1.

#### Loneliness

Experience was longitudinally assessed with a question derived from the Center for Epidemiologic Studies Depression scale (“Do you often feel lonely?”, answering “yes” was defined as presence of loneliness) [17]. Using similar definitions of pattern of change in social isolation, the four patterns of change in loneliness were also ascertained: (1) Never loneliness; (2) Transient loneliness; (3) Incident loneliness; and (4) Persistent loneliness.

### Outcomes

The major outcomes of the current study were the incidence of CVD, all-cause mortality, and CVD mortality. The date and cause of death, including both primary and contributory causes, were obtained through data linkage with the National Health Service (NHS) Information Center of England and Wales and the NHS Central Register of Scotland. Date and hospital diagnoses were determined via record linkage to the Health Episode Statistics of England and Wales and the Scottish Morbidity Records of Scotland. CVD was ascertained using the International Classification of Diseases, Tenth Revision codes–I00-I99. The follow-up of events started from the visit 1 (2012–2013) until incident events, death, or censoring (November 12th, 2021), whichever occurred first.

### CMR Measurements

The UK Biobank imaging study is conducted following standardized pre-defined operating procedures, equipment, and staff training. Between 2014 and 2020, 100,000 participants were recalled to undergo heart, brain, body, and other imaging scans. The details of the CMR protocol in the UK Biobank are provided elsewhere [18]. Briefly, CMR imaging was performed on a clinical wide bore 1.5 Tesla scanner (MAGNETOM Aera, Syngo Platform VD13A, Siemens Healthcare, Erlangen, Germany). Post-processing software cvi (version 5.1.1, Circle Cardiovascular Imaging, Calgary, Canada) was used for the scan analysis. The assessment of cardiac function includes long-axis cines and a complete short-axis stack using balanced steady-state free precession sequence cines. Left ventricular function was automatically derived from CMR data. In the current analysis, we considered the following CMR measures to indicate cardiac function: cardiac index, cardiac output, left ventricular ejection fraction (LVEF), and left ventricular stroke volume (LVSV).

### Covariates

We included the following characteristics assessed at visit 0 as potential confounders [2, 6, 19]. Sociodemographic characteristics included age (continuous, years, calculated from the date of birth), sex (male/female), ethnicity (white/others), marital status (living with partner or not living with partner), current employment status (employed/unemployed), education (college or university degree/non-college or university degree), and Townsend deprivation index (continuous, obtained from the postcode of residence, with a higher score indicating a higher degree of deprivation). Lifestyles and health conditions included smoking status (never/current/past), alcohol consumption frequency (not current/less than three times a week/three or more times a week), physical activity (continuous, metabolic equivalent task - sum of days performing walking, moderate and vigorous activity), TV watching time (continuous, hours/day), healthy diet score (continuous, 0–5 points, calculated from intake amount and frequency of tablespoons, fruit, fish, unprocessed red meat and processed meat), longstanding illnesses, disability, or infirmity (no/yes, collected from the touchscreen questions, self-reported) [3]. More details of the covariates are provided in Supplementary Text S1 and Table S1.

### Statistical Analysis

Descriptive characteristics were reported across patterns of change in social isolation and loneliness. Categorical data are summarized as percentages and continuous data as mean (standard deviation, SD) or median (interquartile range, IQR), when appropriate. Missing data were multiply imputed using the “mice” R package to minimize the potential for selection bias (missing rate < 1%). Notably, the covariate with the highest missing data was physical activity, accounting for 12.50%. Detailed information is provided in Supplementary Table S2.

We used Kaplan-Meier curves to plot the cumulative rate of incident CVD and all-cause and CVD mortality and changes in social isolation and loneliness profile. To estimate the hazard ratio (HR) and 95% confidence interval (CI) of incident CVD and all-cause and CVD mortality for patterns of change in social isolation or loneliness, we used two incremental Cox proportional hazard models [20, 21], with never social isolation or loneliness as the reference group. The proportional hazards assumption for variables was confirmed through examination of the Schoenfeld residual plots for covariates. Model 1 was controlled for age and sex. Model 2 was additionally controlled for ethnicity, current employment status, education, Townsend deprivation index, smoking status, alcohol consumption frequency, physical activity, TV watching time, healthy diet score, longstanding illnesses, disability, or infirmity. We further adjusted for marital status in Model 3. Model 4 was mutually adjusted for social isolation score or loneliness score at baseline based on Model 2. The association between patterns of change in social isolation or loneliness and CMR parameters was investigated using fully adjusted robust regression models [22–25] to address outliers and skewed data. The time interval between visits 1 and 2 (CMR imaging scan) was also included as a potential covariate in robust regression models. In addition, we used functions from the “sandwich” R package to address issues of variance heterogeneity. Furthermore, we incorporated changes in scores of social isolation and loneliness (calculated as the score at follow-up minus the score at baseline) in the model, then evaluated their associations with health outcomes to verify the robustness.

Several sensitivity analyses were performed to examine the robustness of the findings. First, we repeated all the analyses in the complete dataset without missing data and excluded the individuals with depression assessing by the Patient Health Questionnaire-2 (PHQ-2) and hospital inpatient records as previously adopted in the prior studies [26, 27]. Second, we conducted competing risk analyses using a cause-specific and Fine-Gray subdistribution hazards model. Third, we repeated all analyses stratified by age (< 60 and ≥ 60 years) and sex (male/female) to test for robustness and potential variations in different subgroups. Furthermore, cumulative burden (never, once, twice) for social isolation and loneliness was established, ranging from 0 to 2: 0 for never experiencing social isolation or loneliness, 1 for transient or incident cases, and 2 for persistent cases. Associations of the cumulative burden with incident CVD, all-cause mortality, and CVD mortality were then examined. Finally, we created a cumulative score for social isolation and loneliness, summing the scores obtained from three visits to account for the cumulative severity of social isolation and loneliness. To be more specific, during each follow-up, the scores for social isolation ranged from 0 to 3, while loneliness scores ranged from 0 to 1. Consequently, the cumulative score had a potential range of 0 to 9 for social isolation and 0 to 3 for loneliness. We then analyzed the associations between the cumulative score of social isolation and loneliness with CMR functional parameters. A two-sided *P* < 0.05 was considered statistically significant. All statistical analyses were performed using R Statistical Software (version 4.0.2, R Development Core Team, Vienna, Austria).

### Ethical Considerations

The study protocol was approved by the North West Multi-Centre Research Ethics Committee in the UK (Reference numbers: 11/NW/0382). All participants provided written informed consent. The data used in this study were anonymized and de-identified for privacy and confidentiality protection.

## Results

### Characteristics of the Study Sample

The present study included 18,258 individuals (mean [SD] age at baseline, 57.1 [7.4] years; 8892 [48.7%] male) for the main analyses. Compared with participants excluded, those included were more likely to be men and employed, had a higher frequency of alcohol consumption but less smoking, have higher socioeconomic status, and have better health conditions (Supplementary Table S3). An average time interval between visit 0 and visit 1 was about 4.2 (IQR, 3.5 to 4.9) years with minimal slopes measured for social isolation and loneliness at 0.023 and − 0.011, respectively. The characteristics of the study population are summarized in Table 1. The proportions of participants who were never socially isolated and had transient, incident, and persistent social isolation were 81.1%, 5.8%, 6.7%, and 6.4%, respectively. Of the study sample, 80.1% had never reported loneliness, 6.2% experienced transient loneliness, 5.3% experienced incident loneliness, and 8.4% experienced persistent loneliness. Compared with participants who were never socially isolated, those with transient, incident, and persistent social isolation were more likely to be men, employed, and current smokers; had higher education level; lower socioeconomic status; less frequency of alcohol consumption; less physical activity; and worse health conditions. Similar trends were observed among individuals experiencing loneliness, except that they were more likely to be female and had lower education level (Table 1). The average time interval between visits is similar for participants with different change in social isolation and loneliness (data not shown). The characteristics of participants who did and did not participate in visit 1 are displayed in Supplementary Table S4.

**Table 1:** Characteristics of study population by patterns of change in social isolation and loneliness

		Social isolation					Loneliness			
Characteristic	Total (*N* = 18,258)	Never (*N* = 14,801)	Transient (*N* = 1074)	Incident (*N* = 1219)	Persistent (*N* = 1164)		Never (*N* = 14,621)	Transient (*N* = 1136)	Incident (*N* = 966)	Persistent (*N* = 1535)
Age, mean (SD), yrs	57.1 (7.4)	57.4 (7.3)	56.1 (7.7)	56.1 (7.9)	56.0 (7.5)		57.5 (7.3)	55.9 (7.4)	55.6 (7.8)	55.7 (7.6)
Sex, male (%)	8892 (48.7)	7018 (47.4)	573 (53.4)	640 (52.5)	661 (56.8)		7408 (50.7)	466 (41.0)	420 (43.5)	598 (39.0)
Ethnicity, White (%)	17,904 (98.1)	14,541 (98.2)	1041 (96.9)	1195 (98.0)	1127 (96.8)		14,367 (98.3)	1106 (97.4)	945 (97.8)	1486 (96.8)
Currently employed (%)	10,664 (58.4)	8377 (56.6)	744 (69.3)	754 (61.9)	789 (67.8)		8422 (57.6)	699 (61.5)	616 (63.8)	927 (60.4)
College or university degree (%)	8110 (44.4)	6557 (44.3)	491 (45.7)	541 (44.4)	521 (44.8)		6619 (45.3)	482 (42.4)	412 (42.7)	597 (38.9)
Townsend deprivation index^a^	-2.07 (2.66)	-2.23 (2.54)	-1.42 (2.99)	-1.60 (2.95)	-1.13 (3.14)		-2.18 (2.58)	-1.52 (3.00)	-1.78 (2.86)	-1.60 (2.91)
Current smoker (%)	1118 (6.1)	788 (5.3)	95 (8.8)	108 (8.9)	127 (10.9)		837 (5.7)	100 (8.8)	62 (6.4)	119 (7.8)
Alcohol consumption frequency (%)										
Not current	988 (5.4)	745 (5.0)	66 (6.1)	76 (6.2)	101 (8.7)		750 (5.1)	74 (6.5)	53 (5.5)	111 (7.2)
Less than three times a week	8159 (44.7)	6431 (43.4)	510 (47.5)	592 (48.6)	626 (53.8)		6332 (43.3)	578 (50.9)	474 (49.1)	775 (50.5)
Three or more times a week	9111 (49.9)	7625 (51.5)	498 (46.4)	551 (45.2)	437 (37.5)		7539 (51.6)	484 (42.6)	439 (45.4)	649 (42.3)
Physical activity (METs), mean (SD)	10.36 (4.78)	10.61 (4.68)	9.09 (5.07)	9.66 (5.10)	9.12 (5.01)		10.45 (4.75)	10.11 (4.76)	10.09 (4.81)	9.85 (5.05)
TV watching time (hours/day), mean (SD)	2.51 (1.49)	2.48 (1.45)	2.49 (1.63)	2.63 (1.63)	2.69 (1.76)		2.47 (1.44)	2.64 (1.60)	2.48 (1.48)	2.81 (1.81)
Healthy diet score, median [IQR]	3 [2–4]	3 [2–4]	3 [2–4]	3 [2–4]	3 [2–4]		3 [2–4]	3 [2–4]	3 [2–4]	3 [2–4]
Longstanding illnesses, disability, or infirmity (%)	5535 (30.3)	4337 (29.3)	332 (30.9)	427 (35.0)	439 (37.7)		4159 (28.4)	422 (37.1)	328 (34.0)	626 (40.8)
Marital status, live with a partner (%)	13,911 (76.2)	12,224 (82.6)	555 (51.7)	705 (57.8)	427 (36.7)		11,796 (80.7)	623 (54.8)	664 (68.7)	828 (53.9)

^a^Positive values of the index will indicate areas with high material deprivation, whereas those with negative values will indicate relative affluence

IQR: interquartile range; MET: metabolic equivalent of task; SD: standard deviation

### Association of Change in Social Isolation and Loneliness with Subsequent CVD and Mortality

Over a median follow-up of 8.3 (IQR, 8.1 to 8.6) years, 3707 incident CVD events and 856 deaths (310 died from CVD) were recorded from visit 1 (2012–2013). Cumulative rate of incident CVD, all-cause and CVD mortality according to changes in social isolation and loneliness profile was presented in Supplementary Figures S1 and S2, respectively. Among the four patterns of change in social isolation, when taking participants who were never socially isolated as a reference, those with persistent social isolation were at the higher risk for incident CVD (HR 1.17 95% CI 1.03–1.33), all-cause (HR 1.42 95% CI 1.12–1.81), and CVD mortality (HR 1.53 95% CI 1.05–2.23) in the multivariate Cox models adjusted for sociodemographic factors, lifestyle, and health conditions (Model 2, Table 2). The risk of incident CVD among individuals with never social isolation was similar with that of those who with the transient social isolation pattern (HR 1.12 95% CI 0.97–1.28) and incident social isolation pattern (HR 1.03 95% CI 0.90–1.18) (Model 2, Table 2). Similar results were observed for all-cause and CVD mortality rates.

**Table 2:** Associations of patterns of change in social isolation and loneliness with subsequent risk for incident CVD, all-cause mortality, and CVD mortality

	Social isolation					Loneliness			
	Never	Transient	Incident	Persistent		Never	Transient	Incident	Persistent
Incident CVD									
N	11,335	794	886	876		11,227	850	726	1088
Cases/Person-Years	2988/82,369	224/5708	233/6429	262/6215		3000/81,391	212/6243	192/5275	303/7812
Model 1 HR (95% CI)^a^	1.00 [Reference]	1.15 (1.00-1.31)	1.08 (0.95–1.23)	1.25 (1.10–1.41)		1.00 [Reference]	1.05 (0.91–1.20)	1.13 (0.98–1.31)	1.22 (1.08–1.37)
*P* value		0.051	0.259	0.001			0.532	0.092	0.001
Model 2 HR (95% CI)^b^	1.00 [Reference]	1.12 (0.97–1.28)	1.03 (0.90–1.18)	1.17 (1.03–1.33)		1.00 [Reference]	0.98 (0.85–1.12)	1.09 (0.94–1.26)	1.13 (1.00-1.27)
*P* value		0.117	0.640	0.019			0.742	0.249	0.044
**All-cause mortality**									
N	14,801	1074	1219	1164		14,621	1136	966	1535
Cases/Person-Years	658/121,795	58/8793	61/9989	79/9511		666/120,263	56/9329	50/7916	84/12,580
Model 1 HR (95% CI)^a^	1.00 [Reference]	1.30 (1.00-1.70)	1.24 (0.95–1.61)	1.66 (1.31–2.10)		1.00 [Reference]	1.33 (1.01–1.75)	1.42 (1.06–1.89)	1.53 (1.22–1.92)
*P* value		0.054	0.111	< 0.001			0.040	0.017	< 0.001
Model 2 HR (95% CI)^b^	1.00 [Reference]	1.22 (0.93–1.60)	1.10 (0.85–1.44)	1.42 (1.12–1.81)		1.00 [Reference]	1.15 (0.87–1.52)	1.30 (0.97–1.73)	1.28 (1.02–1.61)
*P* value		0.150	0.461	0.004			0.317	0.077	0.036
**CVD mortality**									
N	14,801	1074	1219	1164		14,621	1136	966	1535
Cases/Person-Years	230/123,155	24/8908	21/10,123	35/9650		225/121,679	23/9420	26/7998	36/12,738
Model 1 HR (95% CI)^a^	1.00 [Reference]	1.47 (0.96–2.24)	1.20 (0.77–1.88)	1.92 (1.34–2.77)		1.00 [Reference]	1.69 (1.10–2.60)	2.27 (1.51–3.41)	2.04 (1.43–2.90)
*P* value		0.075	0.424	< 0.001			0.017	< 0.001	< 0.001
Model 2 HR (95% CI)^b^	1.00 [Reference]	1.37 (0.89–2.09)	1.02 (0.65–1.60)	1.53 (1.05–2.23)		1.00 [Reference]	1.34 (0.87–2.07)	1.97 (1.31–2.96)	1.52 (1.06–2.18)
*P* value		0.152	0.923	0.027			0.182	0.001	0.022

^a^Model 1 adjusted for age and sex

^b^Model 2 additionally adjusted for ethnicity, current employment status, education level, Townsend deprivation index, smoking status, alcohol consumption frequency, physical activity, TV watching time, healthy diet score, and longstanding illnesses, disability, or infirmity

CI = confidence interval; CVD: cardiovascular disease; HR: hazard ratio

Likewise, a longer duration of exposure to loneliness was generally associated with a greater risk of developing CVD and mortality (Table 2). Persistent loneliness was independently associated with the risk of subsequent CVD events (HR 1.13 95% CI 1.00–1.27), all-cause (HR 1.28 95% CI 1.02–1.61), and CVD mortality (HR 1.52 95% CI 1.06–2.18) after accounting for covariates including sociodemographic factors, lifestyle, and health conditions (Model 2, Table 2). In line, taking the never loneliness pattern as a reference in the model 2, incident loneliness also remained associated with the risk for CVD mortality (HR 1.97 95% CI 1.31–2.96), but not for incident CVD and all-cause mortality; the association between transient loneliness and all the outcomes attenuated and became insignificant. The associations were attenuated after additionally adjusting marital status and mutually adjusting social isolation score or loneliness score at baseline (Supplementary Model 3&4 Table S5). Score changes of social isolation and loneliness were not significantly associated with subsequent risk for incident CVD and mortality (Supplementary Table S6). However, a linear trend was observed for cumulative burden of social isolation and loneliness (Supplementary Table S7).

The major results were generally robust when the complete dataset was used (Supplementary Table S8), and excluded participants with baseline depression (Supplementary Text S1 and Table S9). Robust results were also found in the Fine–Gray sub-distribution hazard models when accounting for competing risks (Supplementary Table S10). Most of the results of the subgroup analyses were consistent when stratified by age and sex, except the association between persistent social isolation and the risk of CVD mortality among younger and older groups (*P*_interaction_ =0.027) (Supplementary Tables S11 and S12).

### Associations of Change in Social Isolation and Loneliness with Cardiac Function

A subsample of 5188 participants with available CMR data but free of baseline CVD was included to investigate the association between changes in social isolation and loneliness with cardiac function. Compared to participants who were never socially isolated, those with persistent social isolation had a lower LVSV (β=-2.18 95% CI -4.06, -0.30, *P* = 0.023, Table 3). Transient and incident social isolation were not significantly associated with any indicator of cardiac function. Persistent loneliness, rather than transient and incident loneliness, also showed robust associations with lower cardiac function, as indicated by LVSV (β=-2.16, 95% CI -3.75, -0.57, *P* = 0.008), cardiac index (β=-0.09 95% CI -0.14, -0.05, *P* < 0.001), and cardiac output (β=-0.15 95% CI -0.24, -0.05, *P* = 0.002), compared with never loneliness (Table 3). The similar results persisted after additional adjustment for marital status and mutually adjustment for social isolation score or loneliness score at baseline (Supplementary Tables S13 and S14).

**Table 3:** Associations of patterns of change in social isolation and loneliness and their cumulative score with cardiac function by CMR among participants free of CVD

	Transient			Incident			Persistent			Cumulative score	
	β (95% CI)^a^	*P* value		β (95% CI)^a^	*P* value		β (95% CI)^a^	*P* value		β (95% CI)^a^	*P* value
Social isolation											
Cardiac index	0.01 (-0.05, 0.07)	0.813		-0.00 (-0.05, 0.05)	0.927		0.01 (-0.05, 0.06)	0.815		-0.00 (-0.01, 0.00)	0.390
Cardiac output	-0.01 (-0.13, 0.10)	0.808		-0.03 (-0.13, 0.07)	0.600		0.00 (-0.11, 0.11)	0.998		-0.01 (-0.03, 0.00)	0.181
LVEF	-0.07 (-0.76, 0.62)	0.842		-0.32 (-0.97, 0.33)	0.333		-0.43 (-1.08, 0.21)	0.185		-0.07 (-0.16, 0.02)	0.133
LVSV	-1.54 (-3.37, 0.29)	0.100		-1.49 (-3.13, 0.15)	0.075		-2.18 (-4.06, -0.30)	0.023		-0.49 (-0.74, -0.25)	< 0.001
**Loneliness**											
Cardiac index	-0.04 (-0.09, 0.02)	0.179		0.02 (-0.04, 0.08)	0.455		-0.09 (-0.14, -0.05)	< 0.001		-0.03 (-0.04, -0.01)	0.001
Cardiac output	-0.08 (-0.18, 0.03)	0.155		0.04 (-0.08, 0.16)	0.521		-0.15 (-0.24, -0.05)	0.002		-0.04 (-0.07, -0.01)	0.017
LVEF	0.02 (-0.57, 0.62)	0.943		0.54 (-0.14, 1.23)	0.122		-0.38 (-0.95, 0.20)	0.199		-0.09 (-0.27, 0.10)	0.364
LVSV	-0.28 (-2.08, 1.52)	0.761		-0.53 (-2.39, 1.33)	0.577		-2.16 (-3.75, -0.57)	0.008		-0.50 (-1.03, 0.02)	0.059

^a^β were adjusted for age, sex, ethnicity, current employment status, education level, Townsend deprivation index, smoking status, alcohol consumption frequency, physical activity, TV watching time, healthy diet score, and longstanding illnesses, disability, or infirmity. The references here are participants who were never socially isolated or never lonely

CI = confidence interval; CMR: cardiac magnetic resonance; CVD: cardiovascular disease; LVEF: cardiovascular disease; LVSV: left ventricular stroke volume

Leveraging data of social isolation and loneliness across three visits, we conducted robust regression models to investigate the potential dose-response association of the cumulative score of social isolation and loneliness score with subsequent cardiac function. This analysis encompassed measurements of social isolation and loneliness obtained at baseline and two follow-up assessments. As shown in Table 3, a higher cumulative social isolation score was associated with a lower LVSV (β=-0.49 95% CI -0.74, -0.25, *P* < 0.001); a cumulative loneliness score was negatively correlated with cardiac index (β=-0.03, 95% CI -0.04, -0.01, *P* = 0.001), and cardiac output (β=-0.04 95% CI -0.07, -0.01, *P* = 0.017), and LVSV (β=-0.50 95% CI -1.03, -0.02, *P* = 0.059).

Robust results were also found in sensitivity analysis using complete case sample (Supplementary Tables S15-S17). Despite the association of persistent social isolation and loneliness with cardiac dysfunction seemed stronger among men and the youngers (Supplementary Tables S18-S23), the interaction effects with age and sex were insignificant (*P*_interaction_ >0.05).

## Discussion

In this prospective study of the UK Biobank, the novel finding was that the persistence of both social isolation and loneliness over an approximate 4-year exposure period was strongly associated with an elevated risk of incident CVD, all-cause mortality, and CVD mortality. The persistent pattern of changes in social isolation and loneliness was more deleterious than other patterns (never, transient, or incident pattern) regarding cardiovascular and mortality risks and cardiac dysfunction by CMR. In addition, greater cumulative severity of social isolation and loneliness was associated with lower cardiac function. Most observed associations were generally robust in the sensitivity analyses.

To our knowledge, this is the first study to show that persistent social isolation and loneliness are associated with substantially greater risks for both incident CVD and mortality. Although most existing studies have demonstrated that social isolation and loneliness are associated with an increased likelihood of incident CVD or mortality [2–5, 28, 29], the evidence is still inconclusive. Some of the current studies found that baseline social isolation was not associated with incident CVD [19, 30]. Others reported lack of association between loneliness with mortality [16]. One time-point measurement of social disconnection possibly explained this discrepancy in the literature. Our study extends these prior studies by offsetting their common limitations and considering longitudinal changes in social isolation and loneliness using two consecutive measurements. We found that changes in social isolation and loneliness in a persistent pattern were the most deleterious regarding CVD and mortality risks, while incident and transient patterns showed modest risks. Additionally, we identified linear trends in the association of the cumulative burden of social disconnection with incident CVD, all-cause mortality, and CVD mortality. Consistent with our findings, several previous cohort studies reported similar findings regarding dementia risk, where persistent loneliness was the most detrimental pattern for cognitive health [10, 11, 31]. These observations could indicate that social isolation and loneliness had a cumulative effect on cardiovascular and brain health. Collectively, our findings may add evidence supporting the potential causal association between social isolation and loneliness, and the risk of CVD and mortality. Additionally, considering that depression can be both an outcome of social isolation or loneliness [32, 33] and a potential confounding factor [2], we conducted an analysis that excluded participants with a baseline of depression. Our findings revealed that persistent social isolation and loneliness were independent risk factors for all-cause and CVD mortality. Our study also highlighted the association between persistent social isolation and the risk of CVD mortality among younger individuals under 60 years old, which supported the previous findings [28]. The significance lies in the imperative need to understand the underlying mechanisms and implement early interventions for individuals under 60 years old experiencing social isolation and loneliness. Despite the absence of interaction in terms of sex differences, we observed a notable increasing trend in all-cause mortality and CVD mortality among males experiencing social isolation and loneliness. This finding supported prior research [34] suggesting that the lack of social connections may have a more pronounced and adverse impact on men. This phenomenon could potentially be explained by a higher allostatic load and differing effects of inflammation [35] in men experiencing social isolation and loneliness.

The present study also uncovered a potential dose-response association between cumulative social disconnection and poorer cardiac function measured by CMR before the onset of clinical events. This finding corroborates prior clinical and animal studies showing that a higher level of social disconnection was associated with a greater risk of cardiovascular events [2–5], increased blood pressure [36, 37], and accelerated atherogenesis [37]. This result also lend support to the previous hypothesis that psychosocial stress increases the risk of cardiovascular events, possibly by accelerating vascular dysfunction at an earlier stage [38].

The mechanisms by which persistence of social isolation and loneliness may influence cardiovascular and mortality outcomes are complex. Individuals with a longer duration of social isolation and loneliness are more likely to have unhealthy life behaviors, such as smoking, alcohol addiction [39], and physical activity [40]. Lack of medical support or emergency help among individuals who were socially isolated in the long term may also account for a rising mortality risk [2]. Alternatively, several biological links for the observed association have been suggested, including hyperactivation of the hypothalamic-pituitary-adrenal axis and the sympathetic nervous system [41], elevated proinflammatory response [42], and oxidative stress [43]. Once these pathological processes persist following chronic experience of social isolation or loneliness, they are likely to induce subsequent cardiac and vascular dysfunctions, and eventually accelerate the development of CVD and contribute to mortality risk [38].

Our findings should be considered in light of the strengths and limitations of this study. The major strength of the current study is the use of repeated measurements of social isolation and loneliness covering an exposure period of four years, which allows us to track the longitudinal association of change in social isolation and loneliness with the subsequent risk of CVD and mortality. However, this study had some limitations. First, the UK Biobank cohort mainly included participants of white ethnicity; thus, the present findings may not apply to other populations. Second, participants were generally healthier than those who were excluded. This selection bias with healthy volunteer effects may have led to an estimated association towards the null. Third, social isolation and loneliness were screened with several simple questions but incomplete scales in the UK Biobank, which may have led to the underestimation of social isolation and loneliness. However, these questions were adapted from validated scales [15, 44]. Previous studies using the same questions demonstrated comparable hazard risk to those with validated scales [2, 3]. Fourth, it should be acknowledged that there were only two discrete time points for exposure measurement, which could potentially render the incident and transient groups more sensitive to the influence of measurement errors. Fifth, the study was unable to fully consider how changes in certain covariates over time might affect the results because these covariates were measured at different times. This aligns with the approach of existing studies that primarily focused on changes of exposure [10, 11]. Additionally, these covariates have been proven to be stable over time [45], indicating that the changes in covariates are not likely to affect reliability of the current findings. Sixth, the heavy attrition of follow-up data in UK Biobank is indeed a concern for the validity of the present findings. The final sample was highly selected from an already selected original sample, which might introduce bias and limit generalizability. It was important to consider this limitation when interpreting the results and to acknowledge the need for future studies with more complete follow-up data. Seventh, residual confounding was inevitable owing to the observational nature of the study. Only an association, but not causation, can be concluded. Finally, the current results cannot be externally validated in other cohorts.

## Conclusions

In this population-based cohort of the UK Biobank study with repeated measurements of social isolation and loneliness, we documented that compared with other patterns of change, both social isolation and loneliness in a persistent pattern were associated with the greater increase in incident CVD, all-cause and CVD mortality risk, and worse subsequent cardiac function. These findings imply that early implementation of effective and systemic interventions to strengthen social contacts and alleviate feelings of loneliness may bring potential benefits for cardiovascular health and longevity, especially in the post COVID-19 world with the rising pandemic of social disconnection.

## Electronic Supplementary Material

Below is the link to the electronic supplementary material.

Supplementary Material 1

## Data Availability

No datasets were generated or analysed during the current study.

## Abbreviations

CI: Confidence interval
CMR: Cardiovascular magnetic resonance
COVID: 19-Coronavirus disease 2019
CVD: Cardiovascular disease
HR: Hazard ratio
IQR: Interquartile range
LVEF: Left ventricular ejection fraction
LVSV: Left ventricular stroke volume
MET: Metabolic equivalent of task
NHS: National Health Service
SD: Standard deviation
