# Supplementary material for: Evaluation of Changes in Social Isolation and Loneliness with Incident Cardiovascular Events and Mortality
Source: J Epidemiol Glob Health. 2024 May 27;14(3):962–73. doi: 10.1007/s44197-024-00243-3 (PMC11442883; doi:10.1007/s44197-024-00243-3)
Supplement: Supplementary file 1 — Supplementary Material 1 [file 44197_2024_243_MOESM1_ESM.docx]

**Supplementary Material**

**Title: Evaluation of Changes in Social Isolation and Loneliness with Incident Cardiovascular Events and Mortality~~: Prospective Cohort Study~~**

| Supplementary eMethods | | 3 |
| --- | --- | --- |
| Text S1 | Assessment of variables in the UK Biobank | 3 |
| Table S1 | Information about all variables used for adjustment | 4 |
| Table S2 | The number (percentage) of participants with missing covariate data in the analytic sample | 6 |
| Supplementary eResults | | 7 |
| Table S3 | Comparison of characteristics between the total sample and the participants included in the present study in the UK Biobank | 7 |
| Table S4 | Comparison of characteristics between the total sample and the participants who did and did not participate in visit 1 (2012–2013) | 8 |
| Figure S1 | Cumulative rate of incident CVD (A), all-cause mortality (B), and CVD mortality (C) according to changes in social isolation profile | 9 |
| Figure S2 | Cumulative rate of incident CVD (A), all-cause mortality (B), and CVD mortality (C) according to changes in loneliness profile | 10 |
| Table S5 | Associations of patterns of change in social isolation and loneliness with subsequent risk for incident CVD, all-cause mortality, and CVD mortality additionally adjusting marital status and mutually adjusting social isolation score or loneliness score at baseline | 11 |
| Table S6 | Associations of score changes in social isolation and loneliness with subsequent risk for incident CVD, all-cause mortality, and CVD mortality | 12 |
| Table S7 | Associations of patterns of change in cumulative burden of social isolation and loneliness with subsequent risk for incident CVD, all-cause mortality, and CVD mortality | 13 |
| Table S8 | Associations of patterns of change in social isolation and loneliness with subsequent risk for incident CVD, all-cause mortality, and CVD mortality in sample with complete cases | 14 |
| Table S9 | Associations of patterns of change in social isolation and loneliness with subsequent risk for incident CVD, all-cause mortality, and CVD mortality excluded participants with baseline depression | 16 |
| Table S10 | Hazard ratios and 95% CI from the Fine-Gray subdistribution Hazard Models | 18 |
| Table S11 | Associations of patterns of change in social isolation and loneliness with subsequent risk for incident CVD, all-cause mortality, and CVD mortality stratified by age | 19 |
| Table S12 | Associations of patterns of change in social isolation and loneliness with subsequent risk for incident CVD, all-cause mortality, and CVD mortality stratified by sex | 22 |
| Table S13 | Associations of patterns of change in social isolation and loneliness and their cumulative score with cardiac function by CMR among participants free of CVD additionally adjusting marital status | 25 |
| Table S14 | Associations of patterns of change in social isolation and loneliness and their cumulative score with cardiac function by CMR among participants free of CVD mutually adjusting social isolation score or loneliness score at baseline | 26 |
| Table S15 | Associations of patterns of change in social isolation and loneliness with subsequent cardiac function by CMR in sample with complete cases | 27 |
| Table S16 | Associations of patterns of change in social isolation and loneliness with subsequent cardiac function by CMR in sample with complete cases additionally adjusting marital status | 28 |
| Table S17 | Associations of patterns of change in social isolation and loneliness with subsequent cardiac function by CMR in sample with complete cases mutually adjusting social isolation score or loneliness score at baseline | 29 |
| Table S18 | Associations of patterns of change in social isolation and loneliness with subsequent cardiac function by CMR stratified by sex | 30 |
| Table S19 | Associations of patterns of change in social isolation and loneliness with subsequent cardiac function by CMR stratified by sex additionally adjusting marital status | 32 |
| Table S20 | Associations of patterns of change in social isolation and loneliness with subsequent cardiac function by CMR stratified by sex mutually adjusting social isolation score or loneliness score at baseline | 34 |
| Table S21 | Associations of patterns of change in social isolation and loneliness with subsequent cardiac function by CMR stratified by age | 36 |
| Table S22 | Associations of patterns of change in social isolation and loneliness with subsequent cardiac function by CMR stratified by age additionally adjusting marital status | 38 |
| Table S23 | Associations of patterns of change in social isolation and loneliness with subsequent cardiac function by CMR stratified by age mutually adjusting social isolation score or loneliness score at baseline | 40 |

**Supplementary eMethods:**

**Text S1. Assessment of variables in the UK Biobank**

We used self-reported questionnaires, physical-measured variables, and medical history to assess some possible confounders. Age (field ID 21003) was calculated from dates of birth. Sex (female, male; field ID 31), ethnicity (white, others; field ID 21000), marital status (living with partner or not living with partner; field ID 709/6141) and Townsend deprivation index (continuous; a higher score indicates a higher degree of deprivation; field ID 189) which was based on postcode, were selected from the self-reported questionnaires. The following covariate data were obtained from touchscreen questions at the baseline: current employment status (employed, unemployed; field ID 6142), education level (college or university degree, non-college or university degree; field ID 6138), smoking status (never, current, past; field ID 20116), alcohol consumption frequency (not current/less than three times a week/three or more times a week; field ID 1558), physical activity (continuous, metabolic equivalent task-summed days performing walking, moderate and vigorous activity; field ID 22033), TV watching time (continuous, hours/day; field ID 1070), and healthy diet score (continuous, 0-5 points; field ID 1289, 1299, 1309, 1319, 1329, 1339, 1369, 1379, 1389, and 1349).

The healthy diet score was calculated by using the following dietary factors: vegTable Sintake at least four tablespoons each day (median); fruits intake at least three pieces each day (median); fish intake at least twice each week (median); unprocessed red meat intake no more than twice each week (median); and processed meat intake no more than two each week (median). Each point was given for each favourable diet factor, with the total diet score ranging from 0 to 5.

Information on longstanding illnesses, disability, or infirmity were collected from the touchscreen questions at the baseline (field ID 2188). The question 'Do you have any long-standing illness, disability, or infirmity?' allows individuals to respond with 'Yes' or 'No'.

Patient Health Questionnaire-2 (PHQ-2) was used to assess depression (field ID 20510, 20514) at baseline. This 2-item instrument asks about the frequency of depressed mood and anhedonia over the past 2 weeks, with response options being “not at all,” “several days,” “more than half the days,” and “nearly every day,” scored as 0, 1, 2, and 3, respectively. Thus, the PHQ-2 score can range from 0 to 6; a score 3 or greater is indicative of possible depressive disorder. In addition, depression was also assessed by hospital inpatient records (ICD: F32 [single episode depression] and F33 [recurrent depression]).

**Table S1. Information about all variables used for adjustment**

| **Variables** | **Field ID/ ICD-10 codes** | **Description^a^** |
| --- | --- | --- |
| **Exposure** |  |  |
| Social isolation | Field ID 709/1031/6160 | Field ID 709: Number in household  Field ID 1031: Frequency of friend/family visits  Field ID 6160: Leisure/social activities |
| Loneliness | Field ID 2020/2110 | Field ID 2020: Loneliness, isolation  Field ID 2110: Able to confide |
| **Variables in risk-adjusted main analysis** | |  |
| Model 1 adjusted |  |  |
| Age | Field ID 21003 | Age when attended assessment centre |
| Sex | Field ID 31 | Sex |
| Model 2 additionally adjusted |  |  |
| Ethnicity | Field ID 21000 | Ethnic background |
| Current employment status | Field ID 6142 | Current employment status |
| Education level | Field ID 6138 | Qualifications |
| Townsend deprivation index | Field ID 189 | Townsend deprivation index at recruitment |
| Smoking status | Field ID 20116 | Smoking status |
| Alcohol consumption frequency | Field ID 1558 | Alcohol intake frequency |
| Physical activity (MET) | Field ID 22033 | Summed days activity |
| TV watching time | Field ID 1070 | Time spent watching television (TV) |
| Healthy diet score | Field ID 1289/1299/1309/1319/1329/1339/1349/1369/1379/1389 | Field ID 1289: Cooked vegetable intake  Field ID 1299: Salad / raw vegetable intake  Field ID 1309: Fresh fruit intake  Field ID 1319: Dried fruit intake  Field ID 1329: Oily fish intake  Field ID 1339: Non-oily fish intake  Field ID 1349: Processed meat intake  Field ID 1369: Beef intake  Field ID 1379: Lamb/mutton intake  Field ID 1389: Pork intake |
| Longstanding illnesses, disability, or infirmity | Field ID 2188 | Long-standing illness, disability or infirmity |
| Marital status | Field ID 709/6141 | Field ID 709: Number in household  Field ID 6141: How are people in household related to participant |
| **Variables used in the sensitivity analysis** | |  |
| Cardiovascular disease (chronic disease) | ICD I00-I99 |  |
| Depression | Field ID 20510, 20514; ICD F33, F34 | Field ID 20510: Recent feelings of depression  Field ID 20514: Recent lack of interest or pleasure in doing things |

^a^Detailed information is provided on the UK Biobank website: https://biobank.ndph.ox.ac.uk/showcase/search.cgi.

MET=metabolic equivalent of task; ICD-10=International Classification of Diseases, Tenth Revision.

**Table S2. The number (percentage) of participants with missing covariate data in the analytic sample**

| **Variables** | N | % |
| --- | --- | --- |
| Model 1 adjusted |  |  |
| Age | 0 | 0.00 |
| Sex | 0 | 0.00 |
| Model 2 additionally adjusted |  |  |
| Ethnicity | 32 | 0.18 |
| Current employment status | 95 | 0.52 |
| Education level | 59 | 0.32 |
| Townsend deprivation index | 12 | 0.07 |
| Smoking status | 35 | 0.19 |
| Alcohol consumption frequency | 4 | 0.02 |
| Physical activity (MET) | 2288 | 12.50 |
| TV watching time | 45 | 0.25 |
| Healthy diet score | 1 | 0.01 |
| Longstanding illnesses, disability, or infirmity | 286 | 1.57 |
| Marital status | 6 | 0.03 |

MET=metabolic equivalent of task.

**Supplementary eResults**

**Table S3. Comparison of characteristics between the total sample and the participants included in the present study** **in the UK Biobank**

| **Characteristic** | **Total (N=502,505)** | **Included (N=18,258)** | **Excluded (N=484,247)** |
| --- | --- | --- | --- |
| Age, mean (SD), yrs | 57 (8.1) | 57 (7.4) | 57 (8.1) |
| Sex, male (%) | 229,122 (46) | 8892 (49) | 220,230 (46) |
| Ethnicity, White (%) | 472,695 (98) | 17,874 (99) | 454,821 (98) |
| Currently employed (%) | 287,149 (58) | 10,614 (58) | 276,535 (58) |
| College or university degree (%) | 161,163 (33) | 8093 (45) | 153,070 (32) |
| Townsend deprivation index ^a^ | -1.29 (3.10) | -2.07 (2.66) | -1.26 (3.11) |
| Current smoker (%) | 52,978 (11) | 1117 (6) | 51,861 (11) |
| Alcohol consumption frequency (%) |  |  |  |
| Not current | 40,635 (8) | 988 (5) | 39,647 (8) |
| Less than three times a week | 243,131 (49) | 8158 (45) | 234,973 (49) |
| Three or more times a week | 217,194 (43) | 9109 (50) | 208,085 (43) |
| Physical activity (METs), mean (SD) | 10.62 (4.84) | 10.38 (4.77) | 10.63 (4.85) |
| TV watching time (hours/day), mean (SD) | 2.80 (1.68) | 2.50 (1.49) | 2.81 (1.68) |
| Healthy diet score, median [IQR] | 3 [2-4] | 3 [2-4] | 3 [2-4] |
| Longstanding illnesses, disability, or infirmity (%) | 159,897 (33) | 5440 (30) | 154,457 (33) |
| Marital status, live with a partner (%) | 362,607 (72.2) | 13,911 (76.2) | 348,700 (72.0) |

^a^ Positive values of the index will indicate areas with high material deprivation, whereas those with negative values will indicate relative affluence.

IQR=interquartile range; MET=metabolic equivalent of task; SD=standard deviation.

**Table S4. Comparison of characteristics between the total sample and the participants who did and did not participate in visit 1 (2012–2013)**

| **Characteristic** | **Total (N=502,505)** | **Participated (N=20,344)** | **Did not participate (N=482,161)** |
| --- | --- | --- | --- |
| Age, mean (SD), yrs | 57 (8.1) | 57 (7.4) | 57 (8.1) |
| Sex, male (%) | 229,122 (46) | 9,937 (49) | 219,185 (46) |
| Ethnicity, White (%) | 472,695 (98) | 19,851 (99) | 452,844 (98) |
| Currently employed (%) | 287,149 (58) | 11,771 (58) | 275,378 (58) |
| College or university degree (%) | 161,163 (33) | 8669 (44) | 152,494 (32) |
| Townsend deprivation index ^a^ | -1.29 (3.10) | -2.03 (2.69) | -1.26 (3.11) |
| Current smoker (%) | 52,978 (11) | 1283 (6) | 51,695 (11) |
| Alcohol consumption frequency (%) |  |  |  |
| Not current | 40,635 (8) | 1138 (6) | 39,497 (8) |
| Less than three times a week | 243,131 (49) | 9119 (45) | 234,012 (49) |
| Three or more times a week | 217,194 (43) | 10,077 (50) | 207,117 (43) |
| Physical activity (METs), mean (SD) | 10.62 (4.84) | 10.35 (4.78) | 10.63 (4.85) |
| TV watching time (hours/day), mean (SD) | 2.80 (1.68) | 2.51 (1.51) | 2.81 (1.68) |
| Healthy diet score, median [IQR] | 3 [2-4] | 3 [2-4] | 3 [2-4] |
| Longstanding illnesses, disability, or infirmity (%) | 159,897 (33) | 6086 (31) | 153,811 (33) |
| Marital status, live with a partner (%) | 362,607 (72.2) | 15,370 (75.6) | 347,236 (72.0) |

^a^ Positive values of the index will indicate areas with high material deprivation, whereas those with negative values will indicate relative affluence.

IQR=interquartile range; MET=metabolic equivalent of task; SD=standard deviation.


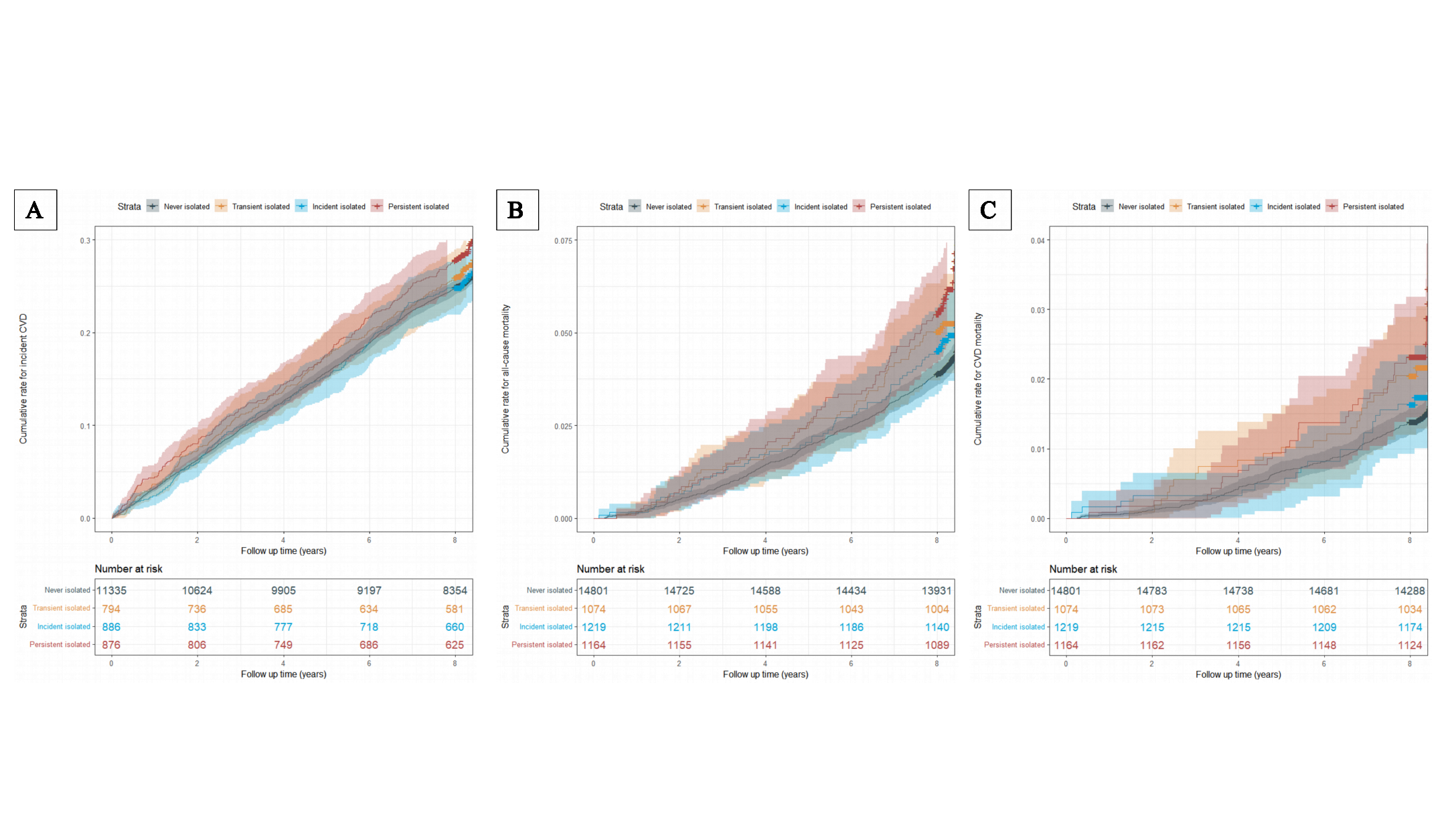
**Figure S1. Cumulative rate of incident CVD (A), all-cause mortality (B), and CVD mortality (C) according to changes in social isolation profile**

CVD=cardiovascular disease.


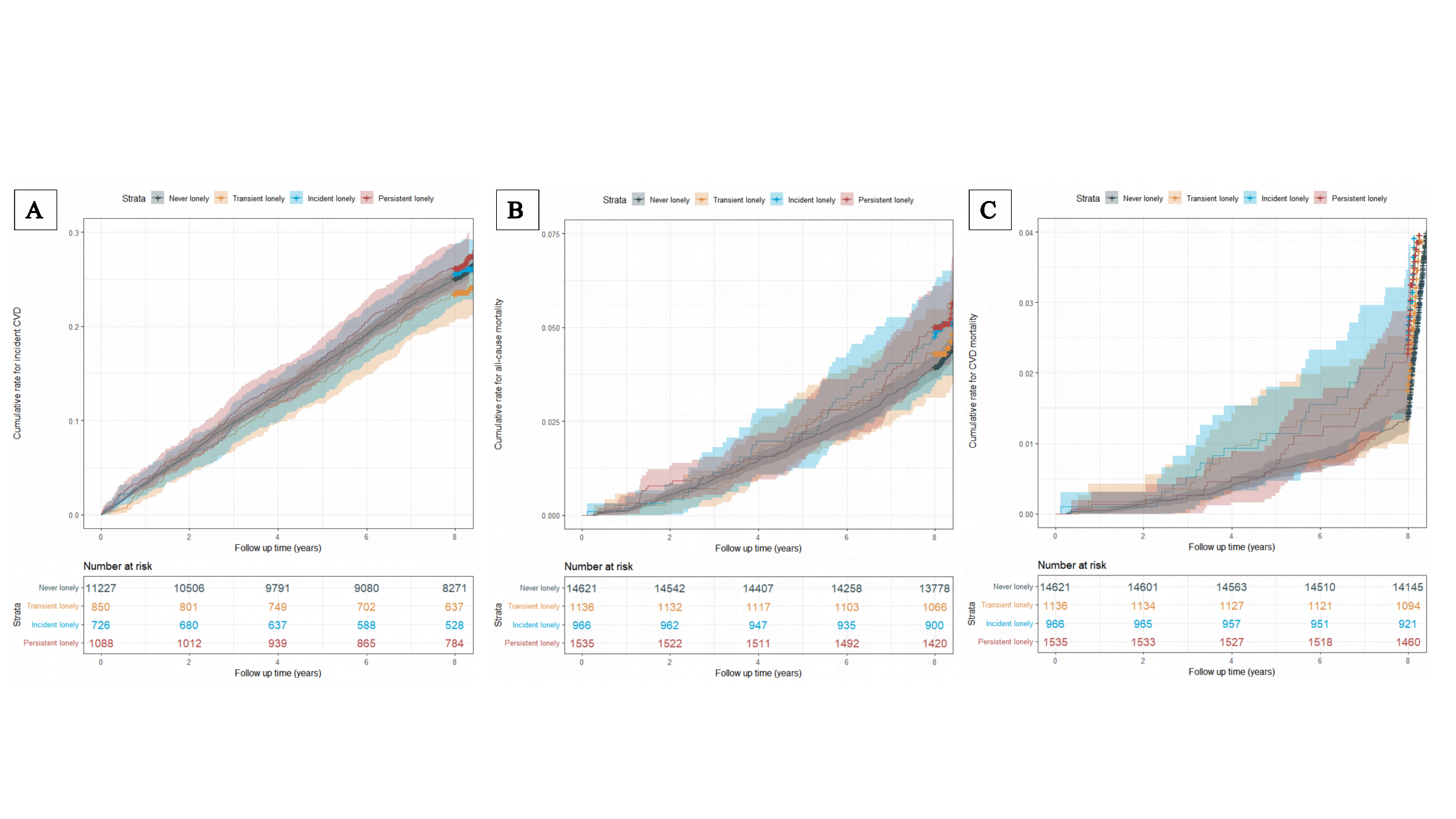
**Figure S2. Cumulative rate of incident CVD (A), all-cause mortality (B), and CVD mortality (C) according to changes in loneliness profile**

CVD=cardiovascular disease.

**Table S5. Associations of patterns of change in social isolation and loneliness with subsequent risk for incident CVD, all-cause mortality, and CVD mortality additionally adjusting marital status** **and mutually adjusting social isolation score or loneliness score at baseline**

|  | **Social isolation** | | | |  | **Loneliness** | | | |
| --- | --- | --- | --- | --- | --- | --- | --- | --- | --- |
|  | **Never** | **Transient** | **Incident** | **Persistent** |  | **Never** | **Transient** | **Incident** | **Persistent** |
| **Incident CVD** |  |  |  |  |  |  |  |  |  |
| **Model 3 HR (95% CI)^a^** | 1.00 [Reference] | 1.14 (0.99-1.31) | 1.05 (0.92-1.20) | 1.20 (1.05-1.38) |  | 1.00 [Reference] | 0.99 (0.86-1.14) | 1.09 (0.94-1.27) | 1.14 (1.01-1.29) |
| ***P* value** |  | 0.068 | 0.487 | 0.007 |  |  | 0.843 | 0.229 | 0.032 |
| **Model 4 HR (95% CI)^b^** | 1.00 [Reference] | 1.11 (0.97-1.28) | 1.03 (0.90-1.18) | 1.16 (1.02-1.32) |  | 1.00 [Reference] | 0.97 (0.84-1.12) | 1.08 (0.94-1.25) | 1.12 (0.99-1.26) |
| ***P* value** |  | 0.121 | 0.652 | 0.022 |  |  | 0.661 | 0.283 | 0.066 |
| **All-cause mortality** |  |  |  |  |  |  |  |  |  |
| **Model 3 HR (95% CI)^a^** | 1.00 [Reference] | 1.14 (0.86-1.50) | 1.05 (0.81-1.38) | 1.29 (1.00-1.66) |  | 1.00 [Reference] | 1.09 (0.82-1.43) | 1.27 (0.95-1.69) | 1.20 (0.95-1.52) |
| ***P* value** |  | 0.365 | 0.706 | 0.049 |  |  | 0.561 | 0.107 | 0.130 |
| **Model 4 HR (95% CI)^b^** | 1.00 [Reference] | 1.20 (0.91-1.57) | 1.09 (0.84-1.42) | 1.38 (1.08-1.76) |  | 1.00 [Reference] | 1.11 (0.84-1.47) | 1.26 (0.95-1.69) | 1.22 (0.97-1.54) |
| ***P* value** |  | 0.191 | 0.526 | 0.010 |  |  | 0.445 | 0.112 | 0.093 |
| **CVD mortality** |  |  |  |  |  |  |  |  |  |
| **Model 3 HR (95% CI)^a^** | 1.00 [Reference] | 1.21 (0.78-1.88) | 0.94 (0.60-1.48) | 1.29 (0.86-1.93) |  | 1.00 [Reference] | 1.23 (0.79-1.91) | 1.91 (1.27-2.88) | 1.38 (0.95-1.99) |
| ***P* value** |  | 0.392 | 0.791 | 0.211 |  |  | 0.352 | 0.002 | 0.088 |
| **Model 4 HR (95% CI)^b^** | 1.00 [Reference] | 1.32 (0.86-2.02) | 1.00 (0.63-1.56) | 1.43 (0.97-2.10) |  | 1.00 [Reference] | 1.29 (0.84-2.00) | 1.91 (1.27-2.88) | 1.43 (1.00-2.06) |
| ***P* value** |  | 0.207 | 0.983 | 0.068 |  |  | 0.246 | 0.002 | 0.053 |

^a^Model 3 adjusted for age, sex, ethnicity, current employment status, education level, Townsend deprivation index, smoking status, alcohol consumption frequency, physical activity, TV watching time, healthy diet score, longstanding illnesses, disability, or infirmity, and marital status.

^b^Model 4 adjusted for age, sex, ethnicity, current employment status, education level, Townsend deprivation index, smoking status, alcohol consumption frequency, physical activity, TV watching time, healthy diet score, longstanding illnesses, disability, or infirmity, and social isolation or loneliness.

CI=confidence interval; CVD=cardiovascular disease; HR=hazard ratio.

**Table S6. Associations of score changes in social isolation and loneliness with subsequent risk for incident CVD, all-cause mortality, and CVD mortality**

|  | **Score changes** | | | | |
| --- | --- | --- | --- | --- | --- |
|  | **Social isolation** | ***P* value** |  | **Loneliness** | ***P* value** |
| **Incident CVD** |  |  |  |  |  |
| **Model 1 HR (95% CI) ^a^** | 1.03 (0.95-1.11) | 0.504 |  | 1.06 (0.95-1.18) | 0.278 |
| **Model 2 HR (95% CI) ^b^** | 1.02 (0.95-1.11) | 0.533 |  | 1.06 (0.96-1.18) | 0.258 |
| **Model 3 HR (95% CI) ^c^** | 1.02 (0.95-1.11) | 0.537 |  | 1.06 (0.95-1.18) | 0.271 |
| **Model 4 HR (95% CI) ^d^** | 1.02 (0.95-1.11) | 0.527 |  | 1.06 (0.96-1.18) | 0.258 |
| **All-cause mortality** |  |  |  |  |  |
| **Model 1 HR (95% CI) ^a^** | 1.01 (0.79-1.29) | 0.925 |  | 0.69 (0.49-0.97) | 0.033 |
| **Model 2 HR (95% CI) ^b^** | 1.01 (0.79-1.29) | 0.935 |  | 0.70 (0.50-0.98) | 0.039 |
| **Model 3 HR (95% CI) ^c^** | 1.02 (0.80-1.30) | 0.898 |  | 0.72 (0.51-1.00) | 0.051 |
| **Model 4 HR (95% CI) ^d^** | 1.01 (0.79-1.29) | 0.930 |  | 0.70 (0.50-0.98) | 0.039 |
| **CVD mortality** |  |  |  |  |  |
| **Model 1 HR (95% CI) ^a^** | 0.89 (0.60-1.33) | 0.567 |  | 0.81 (0.46-1.41) | 0.454 |
| **Model 2 HR (95% CI) ^b^** | 0.87 (0.58-1.30) | 0.492 |  | 0.83 (0.48-1.43) | 0.497 |
| **Model 3 HR (95% CI) ^c^** | 0.88 (0.59-1.31) | 0.526 |  | 0.85 (0.49-1.46) | 0.549 |
| **Model 4 HR (95% CI) ^d^** | 0.87 (0.58-1.30) | 0.497 |  | 0.83 (0.48-1.44) | 0.514 |

^a^Model 1 adjusted for age and sex;

^b^Model 2 additionally adjusted for ethnicity, current employment status, education level, Townsend deprivation index, smoking status, alcohol consumption frequency, physical activity, TV watching time, healthy diet score, and longstanding illnesses, disability, or infirmity.

^c^Model 3 additionally adjusted for marital status.

^d^Model 4 adjusted for age, sex, ethnicity, current employment status, education level, Townsend deprivation index, smoking status, alcohol consumption frequency, physical activity, TV watching time, healthy diet score, longstanding illnesses, disability, or infirmity, and social isolation or loneliness.

CI=confidence interval; CVD=cardiovascular disease; HR=hazard ratio.

**Table** **S7. Associations of patterns of change in cumulative burden of social isolation and loneliness with subsequent risk for incident CVD, all-cause mortality, and CVD mortality**

|  | **Cumulative burden** | | | | |
| --- | --- | --- | --- | --- | --- |
|  | **Social isolation** | ***P* value** |  | **Loneliness** | ***P* value** |
| **Incident CVD** |  |  |  |  |  |
| **Model 1 β (95% CI) ^a^** | 0.11 (0.05-0.16) | <0.001 |  | 0.10 (0.04-0.15) | 0.001 |
| **Model 2 β (95% CI) ^b^** | 0.07 (0.02-0.13) | 0.010 |  | 0.05 (-0.00-0.11) | 0.053 |
| **Model 3 β (95% CI) ^c^** | 0.09 (0.03-0.15) | 0.003 |  | 0.06 (0.00-0.12) | 0.036 |
| **Model 4 β (95% CI) ^d^** | 0.07 (0.02-0.13) | 0.012 |  | 0.05 (-0.01-0.10) | 0.083 |
| **All-cause mortality** |  |  |  |  |  |
| **Model 1 β (95% CI) ^a^** | 0.25 (0.14-0.36) | <0.001 |  | 0.23 (0.13-0.34) | <0.001 |
| **Model 2 β (95% CI) ^b^** | 0.17 (0.06-0.28) | 0.002 |  | 0.14 (0.03-0.24) | 0.011 |
| **Model 3 β (95% CI) ^c^** | 0.12 (-0.00-0.24) | 0.050 |  | 0.10 (-0.01-0.21) | 0.062 |
| **Model 4 β (95% CI) ^d^** | 0.15 (0.04-0.27) | 0.007 |  | 0.11 (0.01-0.22) | 0.040 |
| **CVD mortality** |  |  |  |  |  |
| **Model 1 β (95% CI) ^a^** | 0.32 (0.15-0.49) | <0.001 |  | 0.41 (0.25-0.57) | <0.001 |
| **Model 2 β (95% CI) ^b^** | 0.20 (0.03-0.38) | 0.024 |  | 0.26 (0.09-0.42) | 0.002 |
| **Model 3 β (95% CI) ^c^** | 0.11 (-0.08-0.30) | 0.244 |  | 0.21 (0.04-0.38) | 0.015 |
| **Model 4 β (95% CI) ^d^** | 0.17 (-0.01-0.35) | 0.066 |  | 0.23 (0.06-0.39) | 0.007 |

^a^Model 1 adjusted for age and sex;

^b^Model 2 additionally adjusted for ethnicity, current employment status, education level, Townsend deprivation index, smoking status, alcohol consumption frequency, physical activity, TV watching time, healthy diet score, and longstanding illnesses, disability, or infirmity.

^c^Model 3 additionally adjusted for marital status.

^d^Model 4 adjusted for age, sex, ethnicity, current employment status, education level, Townsend deprivation index, smoking status, alcohol consumption frequency, physical activity, TV watching time, healthy diet score, longstanding illnesses, disability, or infirmity, and social isolation or loneliness.

CI=confidence interval; CVD=cardiovascular disease.

**Table S8. Associations of patterns of change in social isolation and loneliness with subsequent risk for incident CVD, all-cause mortality, and CVD mortality in sample with complete cases**

|  | **Social isolation** | | | |  | **Loneliness** | | | |
| --- | --- | --- | --- | --- | --- | --- | --- | --- | --- |
|  | **Never** | **Transient** | **Incident** | **Persistent** |  | **Never** | **Transient** | **Incident** | **Persistent** |
| **Incident CVD** |  |  |  |  |  |  |  |  |  |
| **N** | 9758 | 696 | 746 | 742 |  | 9697 | 722 | 624 | 899 |
| **Cases/Person-Years** | 2503/71,207 | 200/4999 | 199/5425 | 218/5294 |  | 2535/70,588 | 176/5298 | 163/4549 | 246/6491 |
| **Model 1 HR (95% CI) ^a^** | 1.00 [Reference] | 1.21 (1.05-1.40) | 1.12 (0.97-1.29) | 1.25 (1.09-1.44) |  | 1.00 [Reference] | 1.04 (0.90-1.22) | 1.18 (1.00-1.38) | 1.24 (1.08-1.41) |
| ***P* value** |  | 0.010 | 0.129 | 0.002 |  |  | 0.579 | 0.045 | 0.002 |
| **Model 2 HR (95% CI) ^b^** | 1.00 [Reference] | 1.17 (1.01-1.35) | 1.07 (0.93-1.24) | 1.17 (1.02-1.35) |  | 1.00 [Reference] | 0.97 (0.83-1.14) | 1.14 (0.97-1.34) | 1.14 (1.00-1.31) |
| ***P* value** |  | 0.035 | 0.332 | 0.030 |  |  | 0.736 | 0.099 | 0.046 |
| **Model 3 HR (95% CI) ^c^** | 1.00 [Reference] | 1.20 (1.03-1.39) | 1.10 (0.95-1.27) | 1.21 (1.05-1.41) |  | 1.00 [Reference] | 0.99 (0.84-1.15) | 1.15 (0.98-1.35) | 1.16 (1.01-1.32) |
| ***P* value** |  | 0.018 | 0.221 | 0.010 |  |  | 0.858 | 0.087 | 0.032 |
| **Model 4 HR (95% CI) ^d^** | 1.00 [Reference] | 1.17 (1.01-1.35) | 1.07 (0.93-1.24) | 1.17 (1.01-1.34) |  | 1.00 [Reference] | 0.97 (0.83-1.13) | 1.14 (0.97-1.33) | 1.13 (0.99-1.29) |
| ***P* value** |  | 0.037 | 0.341 | 0.035 |  |  | 0.658 | 0.118 | 0.066 |
| **All-cause mortality** |  |  |  |  |  |  |  |  |  |
| **N** | 12,652 | 923 | 1016 | 982 |  | 12,536 | 957 | 820 | 1260 |
| **Cases/Person-Years** | 536/104,241 | 52/7556 | 55/8320 | 65/8030 |  | 547/103,233 | 47/7856 | 43/6730 | 71/10,328 |
| **Model 1 HR (95% CI) ^a^** | 1.00 [Reference] | 1.44 (1.08-1.91) | 1.40 (1.06-1.85) | 1.66 (1.28-2.15) |  | 1.00 [Reference] | 1.38 (1.02-1.86) | 1.53 (1.12-2.09) | 1.64 (1.28-2.10) |
| ***P* value** |  | 0.013 | 0.018 | <0.001 |  |  | 0.035 | 0.007 | <0.001 |
| **Model 2 HR (95% CI) ^b^** | 1.00 [Reference] | 1.35 (1.01-1.80) | 1.24 (0.94-1.65) | 1.42 (1.09-1.85) |  | 1.00 [Reference] | 1.19 (0.88-1.61) | 1.40 (1.02-1.91) | 1.36 (1.05-1.74) |
| ***P* value** |  | 0.042 | 0.125 | 0.009 |  |  | 0.256 | 0.036 | 0.018 |
| **Model 3 HR (95% CI) ^c^** | 1.00 [Reference] | 1.27 (0.94-1.70) | 1.19 (0.90-1.58) | 1.30 (0.98-1.72) |  | 1.00 [Reference] | 1.12 (0.83-1.52) | 1.36 (1.00-1.86) | 1.27 (0.98-1.64) |
| ***P* value** |  | 0.117 | 0.229 | 0.068 |  |  | 0.455 | 0.054 | 0.068 |
| **Model 4 HR (95% CI) ^d^** | 1.00 [Reference] | 1.32 (0.99-1.76) | 1.23 (0.93-1.62) | 1.36 (1.04-1.78) |  | 1.00 [Reference] | 1.15 (0.85-1.56) | 1.36 (0.99-1.86) | 1.29 (1.00-1.67) |
| ***P* value** |  | 0.063 | 0.156 | 0.025 |  |  | 0.363 | 0.054 | 0.047 |
| **CVD mortality** |  |  |  |  |  |  |  |  |  |
| **N** | 12,652 | 923 | 1016 | 982 |  | 12,536 | 957 | 820 | 1260 |
| **Cases/Person-Years** | 180/105,363 | 20/7660 | 20/8437 | 28/8154 |  | 179/104,407 | 19/7931 | 21/6804 | 29/10,471 |
| **Model 1 HR (95% CI) ^a^** | 1.00 [Reference] | 1.58 (0.99-2.51) | 1.49 (0.94-2.36) | 1.97 (1.31-2.97) |  | 1.00 [Reference] | 1.77 (1.10-2.84) | 2.37 (1.50-3.72) | 2.12 (1.43-3.14) |
| ***P* value** |  | 0.054 | 0.093 | 0.001 |  |  | 0.018 | <0.001 | <0.001 |
| **Model 2 HR (95% CI) ^b^** | 1.00 [Reference] | 1.44 (0.90-2.30) | 1.24 (0.78-1.98) | 1.52 (1.00-2.32) |  | 1.00 [Reference] | 1.38 (0.85-2.22) | 2.02 (1.28-3.19) | 1.54 (1.03-2.30) |
| ***P* value** |  | 0.125 | 0.361 | 0.051 |  |  | 0.193 | 0.003 | 0.035 |
| **Model 3 HR (95% CI) ^c^** | 1.00 [Reference] | 1.27 (0.79-2.05) | 1.13 (0.71-1.82) | 1.27 (0.81-1.99) |  | 1.00 [Reference] | 1.24 (0.76-2.01) | 1.94 (1.23-3.07) | 1.37 (0.91-2.06) |
| ***P* value** |  | 0.326 | 0.608 | 0.299 |  |  | 0.389 | 0.004 | 0.137 |
| **Model 4 HR (95% CI) ^d^** | 1.00 [Reference] | 1.39 (0.87-2.22) | 1.21 (0.76-1.93) | 1.42 (0.92-2.18) |  | 1.00 [Reference] | 1.32 (0.82-2.13) | 1.96 (1.24-3.10) | 1.44 (0.96-2.17) |
| ***P* value** |  | 0.174 | 0.422 | 0.113 |  |  | 0.259 | 0.004 | 0.076 |

^a^ Model 1 adjusted for age and sex;

^b^ Model 2 additionally adjusted for ethnicity, current employment status, education level, Townsend deprivation index, smoking status, alcohol consumption frequency, physical activity, TV watching time, healthy diet score, and longstanding illnesses, disability, or infirmity.

^c^ Model 3 additionally adjusted for marital status.

^d^ Model 4 adjusted for age, sex, ethnicity, current employment status, education level, Townsend deprivation index, smoking status, alcohol consumption frequency, physical activity, TV watching time, healthy diet score, longstanding illnesses, disability, or infirmity, and social isolation or loneliness.

CI=confidence interval; CVD=cardiovascular disease; HR=hazard ratio.

**Table S9. Associations of patterns of change in social isolation and loneliness with subsequent risk for incident CVD, all-cause mortality, and CVD mortality excluded participants with baseline depression**

|  | **Social isolation** | | | |  | **Loneliness** | | | |
| --- | --- | --- | --- | --- | --- | --- | --- | --- | --- |
|  | **Never** | **Transient** | **Incident** | **Persistent** |  | **Never** | **Transient** | **Incident** | **Persistent** |
| **Incident CVD** |  |  |  |  |  |  |  |  |  |
| **N** | 11,116 | 766 | 845 | 838 |  | 11,074 | 814 | 682 | 995 |
| **Cases/Person-Years** | 2923/80,841 | 214/5508 | 222/6134 | 250/5952 |  | 2960/80,309 | 199/5995 | 176/4969 | 274/7161 |
| **Model 1 HR (95% CI) ^a^** | 1.00 [Reference] | 1.13 (0.99-1.30) | 1.07 (0.94-1.23) | 1.24 (1.09-1.41) |  | 1.00 [Reference] | 1.02 (0.89-1.18) | 1.10 (0.94-1.28) | 1.19 (1.05-1.35) |
| ***P* value** |  | 0.076 | 0.315 | 0.001 |  |  | 0.758 | 0.243 | 0.006 |
| **Model 2 HR (95% CI) ^b^** | 1.00 [Reference] | 1.11 (0.96-1.28) | 1.03 (0.90-1.18) | 1.17 (1.02-1.33) |  | 1.00 [Reference] | 0.96 (0.83-1.11) | 1.05 (0.90-1.22) | 1.11 (0.98-1.26) |
| ***P* value** |  | 0.144 | 0.677 | 0.022 |  |  | 0.543 | 0.518 | 0.090 |
| **Model 3 HR (95% CI) ^c^** | 1.00 [Reference] | 1.14 (0.98-1.31) | 1.05 (0.91-1.20) | 1.21 (1.05-1.38) |  | 1.00 [Reference] | 0.97 (0.83-1.12) | 1.06 (0.91-1.23) | 1.13 (0.99-1.28) |
| ***P* value** |  | 0.081 | 0.512 | 0.007 |  |  | 0.643 | 0.482 | 0.066 |
| **Model 4 HR (95% CI) ^d^** | 1.00 [Reference] | 1.11 (0.97-1.28) | 1.03 (0.90-1.18) | 1.17 (1.02-1.34) |  | 1.00 [Reference] | 0.95 (0.82-1.10) | 1.05 (0.90-1.22) | 1.10 (0.97-1.25) |
| ***P* value** |  | 0.140 | 0.663 | 0.021 |  |  | 0.473 | 0.565 | 0.125 |
| **All-cause mortality** |  |  |  |  |  |  |  |  |  |
| **N** | 14,478 | 1031 | 1154 | 1106 |  | 14,393 | 1080 | 904 | 1392 |
| **Cases/Person-Years** | 641/119,122 | 57/8437 | 58/9451 | 76/9032 |  | 655/118,380 | 53/8863 | 48/7399 | 76/11,399 |
| **Model 1 HR (95% CI) ^a^** | 1.00 [Reference] | 1.33 (1.02-1.75) | 1.23 (0.94-1.61) | 1.68 (1.32-2.13) |  | 1.00 [Reference] | 1.33 (1.01-1.76) | 1.45 (1.08-1.95) | 1.51 (1.19-1.92) |
| ***P* value** |  | 0.038 | 0.132 | <0.001 |  |  | 0.046 | 0.013 | 0.001 |
| **Model 2 HR (95% CI) ^b^** | 1.00 [Reference] | 1.26 (0.96-1.66) | 1.11 (0.85-1.46) | 1.47 (1.15-1.87) |  | 1.00 [Reference] | 1.16 (0.87-1.53) | 1.33 (0.99-1.78) | 1.29 (1.01-1.64) |
| ***P* value** |  | 0.096 | 0.443 | 0.002 |  |  | 0.316 | 0.061 | 0.039 |
| **Model 3 HR (95% CI) ^c^** | 1.00 [Reference] | 1.17 (0.89-1.55) | 1.06 (0.81-1.39) | 1.33 (1.03-1.72) |  | 1.00 [Reference] | 1.09 (0.82-1.45) | 1.29 (0.96-1.73) | 1.20 (0.94-1.54) |
| ***P* value** |  | 0.265 | 0.683 | 0.031 |  |  | 0.566 | 0.090 | 0.141 |
| **Model 4 HR (95% CI) ^d^** | 1.00 [Reference] | 1.24 (0.94-1.64) | 1.10 (0.84-1.44) | 1.43 (1.11-1.83) |  | 1.00 [Reference] | 1.11 (0.84-1.47) | 1.29 (0.96-1.73) | 1.22 (0.96-1.56) |
| ***P* value** |  | 0.121 | 0.498 | 0.005 |  |  | 0.473 | 0.096 | 0.108 |
| **CVD mortality** |  |  |  |  |  |  |  |  |  |
| **N** | 14,478 | 1031 | 1154 | 1106 |  | 14,393 | 1080 | 904 | 1392 |
| **Cases/Person-Years** | 222/120,466 | 23/8552 | 20/9580 | 33/9167 |  | 221/119,782 | 21/8955 | 24/7481 | 32/11,548 |
| **Model 1 HR (95% CI) ^a^** | 1.00 [Reference] | 1.48 (0.96-2.27) | 1.20 (0.76-1.90) | 1.92 (1.32-2.80) |  | 1.00 [Reference] | 1.63 (1.04-2.56) | 2.23 (1.46-3.41) | 1.99 (1.37-2.89) |
| ***P* value** |  | 0.078 | 0.427 | 0.001 |  |  | 0.033 | <0.001 | <0.001 |
| **Model 2 HR (95% CI) ^b^** | 1.00 [Reference] | 1.39 (0.90-2.15) | 1.05 (0.66-1.66) | 1.59 (1.08-2.34) |  | 1.00 [Reference] | 1.32 (0.84-2.07) | 1.95 (1.27-2.98) | 1.54 (1.06-2.25) |
| ***P* value** |  | 0.137 | 0.842 | 0.019 |  |  | 0.230 | 0.002 | 0.024 |
| **Model 3 HR (95% CI) ^c^** | 1.00 [Reference] | 1.22 (0.78-1.92) | 0.96 (0.60-1.53) | 1.33 (0.88-2.01) |  | 1.00 [Reference] | 1.20 (0.76-1.90) | 1.88 (1.23-2.87) | 1.39 (0.94-2.04) |
| ***P* value** |  | 0.375 | 0.860 | 0.171 |  |  | 0.433 | 0.004 | 0.097 |
| **Model 4 HR (95% CI) ^d^** | 1.00 [Reference] | 1.34 (0.87-2.08) | 1.02 (0.64-1.62) | 1.49 (1.01-2.22) |  | 1.00 [Reference] | 1.26 (0.80-1.98) | 1.88 (1.23-2.88) | 1.45 (0.99-2.12) |
| ***P* value** |  | 0.185 | 0.933 | 0.046 |  |  | 0.318 | 0.004 | 0.057 |

^a^ Model 1 adjusted for age and sex;

^b^ Model 2 additionally adjusted for ethnicity, current employment status, education level, Townsend deprivation index, smoking status, alcohol consumption frequency, physical activity, TV watching time, healthy diet score, and longstanding illnesses, disability, or infirmity.

^c^ Model 3 additionally adjusted for marital status.

^d^ Model 4 adjusted for age, sex, ethnicity, current employment status, education level, Townsend deprivation index, smoking status, alcohol consumption frequency, physical activity, TV watching time, healthy diet score, longstanding illnesses, disability, or infirmity, and social isolation or loneliness.

CI=confidence interval; CVD=cardiovascular disease; HR=hazard ratio.

**Table S10. Hazard ratios and 95% CI from the Fine-Gray subdistribution Hazard Models**

|  | **Social isolation** | | | |  | **Loneliness** | | | |
| --- | --- | --- | --- | --- | --- | --- | --- | --- | --- |
|  | **Never** | **Transient** | **Incident** | **Persistent** |  | **Never** | **Transient** | **Incident** | **Persistent** |
| **Incident CVD** |  |  |  |  |  |  |  |  |  |
| **Model 1 sHR (95% CI) ^a^** | 1.00 [Reference] | 1.15 (1.00-1.32) | 1.08 (0.94-1.23) | 1.24 (1.09-1.41) |  | 1.00 [Reference] | 1.05 (0.91-1.21) | 1.13 (0.98-1.31) | 1.21 (1.08-1.37) |
| ***P* value** |  | 0.046 | 0.270 | 0.001 |  |  | 0.490 | 0.091 | 0.002 |
| **Model 2 sHR (95% CI) ^b^** | 1.00 [Reference] | 1.12 (0.98-1.29) | 1.03 (0.90-1.18) | 1.16 (1.02-1.32) |  | 1.00 [Reference] | 0.98 (0.85-1.13) | 1.09 (0.94-1.26) | 1.13 (1.00-1.27) |
| ***P* value** |  | 0.100 | 0.660 | 0.025 |  |  | 0.810 | 0.240 | 0.053 |
| **Model 3 sHR (95% CI) ^c^** | 1.00 [Reference] | 1.14 (1.00-1.32) | 1.05 (0.91-1.20) | 1.20 (1.05-1.37) |  | 1.00 [Reference] | 0.99 (0.86-1.14) | 1.10 (0.95-1.27) | 1.14 (1.01-1.28) |
| ***P* value** |  | 0.058 | 0.500 | 0.009 |  |  | 0.920 | 0.220 | 0.037 |
| **Model 4 sHR (95% CI) ^d^** | 1.00 [Reference] | 1.14 (0.99-1.32) | 1.05 (0.91-1.20) | 1.19 (1.04-1.37) |  | 1.00 [Reference] | 0.99 (0.86-1.14) | 1.09 (0.94-1.26) | 1.13 (1.00-1.28) |
| ***P* value** |  | 0.059 | 0.510 | 0.011 |  |  | 0.890 | 0.250 | 0.047 |
| **CVD mortality** |  |  |  |  |  |  |  |  |  |
| **Model 1 sHR (95% CI) ^a^** | 1.00 [Reference] | 1.48 (0.98-2.25) | 1.20 (0.77-1.87) | 1.96 (1.37-2.82) |  | 1.00 [Reference] | 1.68 (1.09-2.59) | 2.25 (1.50-3.38) | 2.01 (1.41-2.86) |
| ***P* value** |  | 0.064 | 0.430 | <0.001 |  |  | 0.018 | <0.001 | <0.001 |
| **Model 2 sHR (95% CI) ^b^** | 1.00 [Reference] | 1.36 (0.89-2.07) | 1.02 (0.66-1.59) | 1.55 (1.07-2.25) |  | 1.00 [Reference] | 1.33 (0.86-2.06) | 1.96 (1.31-2.96) | 1.51 (1.06-2.15) |
| ***P* value** |  | 0.160 | 0.920 | 0.019 |  |  | 0.190 | 0.001 | 0.024 |
| **Model 3 sHR (95% CI) ^c^** | 1.00 [Reference] | 1.20 (0.77-1.86) | 0.94 (0.60-1.47) | 1.30 (0.87-1.95) |  | 1.00 [Reference] | 1.22 (0.79-1.89) | 1.90 (1.26-2.86) | 1.36 (0.95-1.95) |
| ***P* value** |  | 0.420 | 0.790 | 0.190 |  |  | 0.370 | 0.002 | 0.094 |
| **Model 4 sHR (95% CI) ^d^** | 1.00 [Reference] | 1.18 (0.76-1.83) | 0.93 (0.59-1.45) | 1.27 (0.85-1.89) |  | 1.00 [Reference] | 1.22 (0.78-1.88) | 1.88 (1.25-2.83) | 1.35 (0.94-1.93) |
| ***P* value** |  | 0.450 | 0.750 | 0.250 |  |  | 0.380 | 0.002 | 0.110 |

^a^ Model 1 adjusted for age and sex;

^b^ Model 2 additionally adjusted for ethnicity, current employment status, education level, Townsend deprivation index, smoking status, alcohol consumption frequency, physical activity, TV watching time, healthy diet score, and longstanding illnesses, disability, or infirmity.

^c^ Model 3 additionally adjusted for marital status.

^d^ Model 4 adjusted for age, sex, ethnicity, current employment status, education level, Townsend deprivation index, smoking status, alcohol consumption frequency, physical activity, TV watching time, healthy diet score, longstanding illnesses, disability, or infirmity, and social isolation or loneliness.

CI=confidence interval; CVD=cardiovascular disease; sHR=subdistribution hazard ratio.

**Table S11. Associations of patterns of change in social isolation and loneliness with subsequent risk** **for incident CVD, all-cause mortality, and CVD mortality stratified by age**

|  | **Social isolation** | | | |  | **Loneliness** | | | |
| --- | --- | --- | --- | --- | --- | --- | --- | --- | --- |
|  | **Never** | **Transient** | **Incident** | **Persistent** |  | **Never** | **Transient** | **Incident** | **Persistent** |
| **Incident CVD** |  |  |  |  |  |  |  |  |  |
| **<60 years** |  |  |  |  |  |  |  |  |  |
| **N** | 6686 | 537 | 594 | 591 |  | 6596 | 587 | 491 | 734 |
| **Cases/Person-Years** | 1298/50,577 | 118/4055 | 116/4499 | 139/4371 |  | 1284/49,985 | 122/4419 | 106/3676 | 159/5422 |
| **Model 1 HR (95% CI) ^a^** | 1.00 [Reference] | 1.11 (0.92-1.34) | 1.04 (0.86-1.26) | 1.27 (1.06-1.51) |  | 1.00 [Reference] | 1.13 (0.94-1.37) | 1.22 (1.00-1.49) | 1.25 (1.06-1.48) |
| ***P* value** |  | 0.283 | 0.691 | 0.008 |  |  | 0.191 | 0.051 | 0.008 |
| **Model 2 HR (95% CI) ^b^** | 1.00 [Reference] | 1.06 (0.88-1.29) | 0.97 (0.80-1.17) | 1.15 (0.96-1.37) |  | 1.00 [Reference] | 1.01 (0.84-1.22) | 1.16 (0.95-1.41) | 1.13 (0.95-1.33) |
| ***P* value** |  | 0.542 | 0.727 | 0.138 |  |  | 0.910 | 0.151 | 0.161 |
| **Model 3 HR (95% CI) ^c^** | 1.00 [Reference] | 1.07 (0.88-1.30) | 0.98 (0.81-1.19) | 1.17 (0.97-1.41) |  | 1.00 [Reference] | 1.02 (0.84-1.23) | 1.16 (0.95-1.42) | 1.14 (0.96-1.35) |
| ***P* value** |  | 0.470 | 0.822 | 0.099 |  |  | 0.843 | 0.143 | 0.137 |
| **Model 4 HR (95% CI) ^d^** | 1.00 [Reference] | 1.05 (0.81-1.35) | 0.96 (0.79-1.17) | 1.13 (0.88-1.45) |  | 1.00 [Reference] | 1.06 (0.82-1.35) | 1.16 (0.95-1.42) | 1.18 (0.93-1.50) |
| ***P* value** |  | 0.709 | 0.712 | 0.330 |  |  | 0.667 | 0.143 | 0.175 |
| **≥60 years** |  |  |  |  |  |  |  |  |  |
| **N** | 4649 | 257 | 292 | 285 |  | 4631 | 263 | 235 | 354 |
| **Cases/Person-Years** | 1690/31,792 | 106/1653 | 117/1930 | 123/1844 |  | 1716/31,406 | 90/1824 | 86/1598 | 144/2390 |
| **Model 1 HR (95% CI) ^a^** | 1.00 [Reference] | 1.18 (0.97-1.43) | 1.12 (0.93-1.35) | 1.22 (1.02-1.46) |  | 1.00 [Reference] | 0.95 (0.77-1.17) | 1.04 (0.84-1.29) | 1.18 (0.99-1.40) |
| ***P* value** |  | 0.106 | 0.234 | 0.034 |  |  | 0.628 | 0.711 | 0.062 |
| **Model 2 HR (95% CI) ^b^** | 1.00 [Reference] | 1.17 (0.96-1.42) | 1.08 (0.90-1.31) | 1.16 (0.97-1.40) |  | 1.00 [Reference] | 0.93 (0.75-1.15) | 1.00 (0.81-1.25) | 1.11 (0.94-1.32) |
| ***P* value** |  | 0.121 | 0.409 | 0.113 |  |  | 0.519 | 0.974 | 0.228 |
| **Model 3 HR (95% CI) ^c^** | 1.00 [Reference] | 1.21 (0.99-1.48) | 1.10 (0.91-1.34) | 1.21 (1.00-1.48) |  | 1.00 [Reference] | 0.94 (0.76-1.17) | 1.01 (0.81-1.25) | 1.12 (0.94-1.34) |
| ***P* value** |  | 0.068 | 0.305 | 0.051 |  |  | 0.589 | 0.943 | 0.189 |
| **Model 4 HR (95% CI) ^d^** | 1.00 [Reference] | 1.24 (0.97-1.60) | 1.10 (0.91-1.33) | 1.24 (0.97-1.59) |  | 1.00 [Reference] | 0.94 (0.73-1.21) | 1.00 (0.81-1.25) | 1.12 (0.90-1.40) |
| ***P* value** |  | 0.092 | 0.332 | 0.092 |  |  | 0.644 | 0.973 | 0.306 |
| ***P* _interaction_ ^e^** |  |  |  | 0.495 |  |  |  |  | 0.640 |
| **All-cause mortality** |  |  |  |  |  |  |  |  |  |
| **<60 years** |  |  |  |  |  |  |  |  |  |
| **N** | 7966 | 673 | 739 | 729 |  | 7835 | 714 | 601 | 957 |
| **Cases/Person-Years** | 167/66,214 | 20/5568 | 16/6130 | 32/6002 |  | 163/65,123 | 25/5903 | 20/4971 | 27/7918 |
| **Model 1 HR (95% CI) ^a^** | 1.00 [Reference] | 1.49 (0.94-2.37) | 1.14 (0.68-1.90) | 2.25 (1.54-3.29) |  | 1.00 [Reference] | 1.91 (1.25-2.91) | 1.87 (1.17-2.98) | 1.59 (1.06-2.40) |
| ***P* value** |  | 0.093 | 0.621 | <0.001 |  |  | 0.003 | 0.009 | 0.026 |
| **Model 2 HR (95% CI) ^b^** | 1.00 [Reference] | 1.28 (0.80-2.05) | 0.98 (0.59-1.65) | 1.74 (1.17-2.58) |  | 1.00 [Reference] | 1.56 (1.02-2.40) | 1.66 (1.04-2.65) | 1.28 (0.84-1.94) |
| ***P* value** |  | 0.307 | 0.954 | 0.006 |  |  | 0.040 | 0.033 | 0.246 |
| **Model 3 HR (95% CI) ^c^** | 1.00 [Reference] | 1.16 (0.72-1.87) | 0.90 (0.53-1.52) | 1.50 (0.99-2.27) |  | 1.00 [Reference] | 1.43 (0.93-2.21) | 1.63 (1.02-2.60) | 1.16 (0.76-1.78) |
| ***P* value** |  | 0.542 | 0.703 | 0.057 |  |  | 0.104 | 0.042 | 0.483 |
| **Model 4 HR (95% CI) ^d^** | 1.00 [Reference] | 0.84 (0.44-1.58) | 0.90 (0.53-1.52) | 1.11 (0.60-2.03) |  | 1.00 [Reference] | 1.50 (0.84-2.68) | 1.66 (1.04-2.65) | 1.22 (0.67-2.24) |
| ***P* value** |  | 0.582 | 0.700 | 0.744 |  |  | 0.170 | 0.035 | 0.518 |
| **≥60 years** |  |  |  |  |  |  |  |  |  |
| **N** | 6835 | 401 | 480 | 435 |  | 6786 | 422 | 365 | 578 |
| **Cases/Person-Years** | 491/55,581 | 38/3225 | 45/3859 | 47/3510 |  | 503/55,140 | 31/3426 | 30/2945 | 57/4663 |
| **Model 1 HR (95% CI) ^a^** | 1.00 [Reference] | 1.24 (0.89-1.73) | 1.29 (0.95-1.75) | 1.43 (1.06-1.93) |  | 1.00 [Reference] | 1.08 (0.75-1.56) | 1.24 (0.85-1.79) | 1.52 (1.15-2.00) |
| ***P* value** |  | 0.201 | 0.107 | 0.019 |  |  | 0.665 | 0.261 | 0.003 |
| **Model 2 HR (95% CI) ^b^** | 1.00 [Reference] | 1.20 (0.86-1.67) | 1.16 (0.85-1.58) | 1.27 (0.93-1.72) |  | 1.00 [Reference] | 0.95 (0.66-1.37) | 1.15 (0.79-1.66) | 1.29 (0.97-1.70) |
| ***P* value** |  | 0.292 | 0.349 | 0.128 |  |  | 0.789 | 0.473 | 0.076 |
| **Model 3 HR (95% CI) ^c^** | 1.00 [Reference] | 1.13 (0.80-1.60) | 1.12 (0.82-1.53) | 1.18 (0.85-1.63) |  | 1.00 [Reference] | 0.91 (0.63-1.32) | 1.12 (0.78-1.63) | 1.22 (0.92-1.63) |
| ***P* value** |  | 0.475 | 0.472 | 0.318 |  |  | 0.613 | 0.539 | 0.163 |
| **Model 4 HR (95% CI) ^d^** | 1.00 [Reference] | 1.10 (0.71-1.69) | 1.13 (0.82-1.55) | 1.16 (0.75-1.78) |  | 1.00 [Reference] | 0.86 (0.56-1.33) | 1.14 (0.79-1.65) | 1.16 (0.80-1.68) |
| ***P* value** |  | 0.671 | 0.445 | 0.506 |  |  | 0.499 | 0.495 | 0.430 |
| ***P* _interaction_ ^e^** |  |  |  | 0.357 |  |  |  |  | 0.143 |
| **CVD mortality** |  |  |  |  |  |  |  |  |  |
| **<60 years** |  |  |  |  |  |  |  |  |  |
| **N** | 7966 | 673 | 739 | 729 |  | 7835 | 714 | 601 | 957 |
| **Cases/Person-Years** | 50/66,576 | 8/5613 | 4/6179 | 19/6044 |  | 51/65,486 | 7/5944 | 12/4998 | 11/7983 |
| **Model 1 HR (95% CI) ^a^** | 1.00 [Reference] | 1.96 (0.93-4.14) | 0.93 (0.33-2.57) | 4.33 (2.54-7.37) |  | 1.00 [Reference] | 1.74 (0.79-3.85) | 3.64 (1.93-6.85) | 2.13 (1.11-4.11) |
| ***P* value** |  | 0.078 | 0.886 | <0.001 |  |  | 0.169 | <0.001 | 0.023 |
| **Model 2 HR (95% CI) ^b^** | 1.00 [Reference] | 1.72 (0.80-3.68) | 0.74 (0.27-2.08) | 2.94 (1.66-5.20) |  | 1.00 [Reference] | 1.24 (0.55-2.75) | 3.00 (1.59-5.69) | 1.40 (0.71-2.76) |
| ***P* value** |  | 0.164 | 0.571 | <0.001 |  |  | 0.605 | 0.001 | 0.326 |
| **Model 3 HR (95% CI) ^c^** | 1.00 [Reference] | 1.59 (0.73-3.46) | 0.69 (0.24-1.95) | 2.63 (1.43-4.84) |  | 1.00 [Reference] | 1.12 (0.50-2.51) | 2.97 (1.57-5.63) | 1.25 (0.63-2.49) |
| ***P* value** |  | 0.241 | 0.483 | 0.002 |  |  | 0.783 | 0.001 | 0.522 |
| **Model 4 HR (95% CI) ^d^** | 1.00 [Reference] | 1.57 (0.53-4.66) | 0.73 (0.26-2.06) | 2.68 (0.98-7.27) |  | 1.00 [Reference] | 0.99 (0.36-2.68) | 2.96 (1.56-5.62) | 1.08 (0.41-2.84) |
| ***P* value** |  | 0.417 | 0.552 | 0.054 |  |  | 0.979 | 0.001 | 0.870 |
| **≥60 years** |  |  |  |  |  |  |  |  |  |
| **N** | 6835 | 401 | 480 | 435 |  | 6786 | 422 | 365 | 578 |
| **Cases/Person-Years** | 180/56,579 | 16/3295 | 17/3945 | 16/3606 |  | 174/56,193 | 16/3476 | 14/2999 | 25/4755 |
| **Model 1 HR (95% CI) ^a^** | 1.00 [Reference] | 1.31 (0.78-2.19) | 1.30 (0.79-2.14) | 1.17 (0.69-1.98) |  | 1.00 [Reference] | 1.68 (1.01-2.81) | 1.73 (1.00-2.98) | 2.02 (1.32-3.08) |
| ***P* value** |  | 0.314 | 0.297 | 0.572 |  |  | 0.047 | 0.050 | 0.001 |
| **Model 2 HR (95% CI) ^b^** | 1.00 [Reference] | 1.27 (0.75-2.13) | 1.13 (0.69-1.87) | 0.95 (0.55-1.64) |  | 1.00 [Reference] | 1.38 (0.82-2.33) | 1.56 (0.90-2.70) | 1.58 (1.03-2.42) |
| ***P* value** |  | 0.374 | 0.629 | 0.844 |  |  | 0.221 | 0.112 | 0.038 |
| **Model 3 HR (95% CI) ^c^** | 1.00 [Reference] | 1.10 (0.64-1.87) | 1.03 (0.62-1.72) | 0.78 (0.44-1.38) |  | 1.00 [Reference] | 1.28 (0.76-2.17) | 1.51 (0.87-2.62) | 1.45 (0.93-2.25) |
| ***P* value** |  | 0.732 | 0.897 | 0.388 |  |  | 0.360 | 0.139 | 0.097 |
| **Model 4 HR (95% CI) ^d^** | 1.00 [Reference] | 0.93 (0.47-1.83) | 1.04 (0.62-1.74) | 0.67 (0.32-1.40) |  | 1.00 [Reference] | 1.20 (0.64-2.27) | 1.55 (0.89-2.68) | 1.36 (0.77-2.42) |
| ***P* value** |  | 0.831 | 0.886 | 0.286 |  |  | 0.566 | 0.120 | 0.295 |
| ***P* _interaction_ ^e^** |  |  |  | 0.027 |  |  |  |  | 0.117 |

^a^Model 1 adjusted for age and sex;

^b^Model 2 adjusted for age, sex, ethnicity, current employment status, education level, Townsend deprivation index, smoking status, alcohol consumption frequency, physical activity, TV watching time, healthy diet score, and longstanding illnesses, disability, or infirmity.

^c^Model 3 adjusted for age, sex, ethnicity, current employment status, education level, Townsend deprivation index, smoking status, alcohol consumption frequency, physical activity, TV watching time, healthy diet score, longstanding illnesses, disability, or infirmity, and marital status.

^d^Model 4 adjusted for age, sex, ethnicity, current employment status, education level, Townsend deprivation index, smoking status, alcohol consumption frequency, physical activity, TV watching time, healthy diet score, longstanding illnesses, disability, or infirmity, and social isolation or loneliness.

^e^*P* _interaction_ was calculated after adjusting for age*social isolation change or loneliness change, sex, ethnicity, current employment status, education level, Townsend deprivation index, smoking status, alcohol consumption frequency, physical activity, TV watching time, healthy diet score, and longstanding illnesses, disability, or infirmity.

CI=confidence interval; CVD=cardiovascular disease; HR=hazard ratio.

**Table S12. Associations of patterns of change in social isolation and loneliness with subsequent risk for incident CVD, all-cause mortality, and CVD mortality stratified by sex**

|  | **Social isolation** | | | |  | **Loneliness** | | | |
| --- | --- | --- | --- | --- | --- | --- | --- | --- | --- |
|  | **Never** | **Transient** | **Incident** | **Persistent** |  | **Never** | **Transient** | **Incident** | **Persistent** |
| **Incident CVD** |  |  |  |  |  |  |  |  |  |
| **Men** |  |  |  |  |  |  |  |  |  |
| **N** | 5101 | 404 | 447 | 466 |  | 5408 | 316 | 303 | 391 |
| **Cases/Person-Years** | 1583/35,917 | 127/2830 | 133/3170 | 148/3293 |  | 1680/38,087 | 100/2232 | 88/2184 | 123/2705 |
| **Model 1 HR (95% CI) ^a^** | 1.00 [Reference] | 1.10 (0.92-1.32) | 1.06 (0.89-1.27) | 1.13 (0.95-1.34) |  | 1.00 [Reference] | 1.17 (0.95-1.43) | 1.08 (0.87-1.33) | 1.19 (0.99-1.42) |
| ***P* value** |  | 0.295 | 0.488 | 0.160 |  |  | 0.138 | 0.509 | 0.069 |
| **Model 2 HR (95% CI) ^b^** | 1.00 [Reference] | 1.08 (0.90-1.29) | 1.02 (0.85-1.21) | 1.06 (0.89-1.26) |  | 1.00 [Reference] | 1.10 (0.90-1.35) | 1.05 (0.84-1.30) | 1.11 (0.92-1.34) |
| ***P* value** |  | 0.432 | 0.861 | 0.493 |  |  | 0.345 | 0.687 | 0.259 |
| **Model 3 HR (95% CI) ^c^** | 1.00 [Reference] | 1.06 (0.88-1.27) | 1.00 (0.84-1.20) | 1.03 (0.86-1.24) |  | 1.00 [Reference] | 1.09 (0.89-1.34) | 1.04 (0.84-1.29) | 1.10 (0.91-1.32) |
| ***P* value** |  | 0.546 | 0.958 | 0.718 |  |  | 0.425 | 0.718 | 0.343 |
| **Model 4 HR (95% CI) ^d^** | 1.00 [Reference] | 1.03 (0.81-1.31) | 1.01 (0.84-1.21) | 1.02 (0.80-1.29) |  | 1.00 [Reference] | 1.03 (0.81-1.31) | 1.04 (0.84-1.29) | 1.03 (0.82-1.30) |
| ***P* value** |  | 0.792 | 0.938 | 0.892 |  |  | 0.813 | 0.722 | 0.796 |
| **Women** |  |  |  |  |  |  |  |  |  |
| **N** | 6234 | 390 | 439 | 410 |  | 5819 | 534 | 423 | 697 |
| **Cases/Person-Years** | 1405/46,453 | 97/2878 | 100/3259 | 114/2921 |  | 1320/43,304 | 112/4011 | 104/3091 | 180/5106 |
| **Model 1 HR (95% CI) ^a^** | 1.00 [Reference] | 1.20 (0.97-1.47) | 1.10 (0.90-1.35) | 1.43 (1.18-1.74) |  | 1.00 [Reference] | 0.95 (0.78-1.16) | 1.19 (0.97-1.45) | 1.24 (1.06-1.45) |
| ***P* value** |  | 0.090 | 0.365 | <0.001 |  |  | 0.622 | 0.091 | 0.007 |
| **Model 2 HR (95% CI) ^b^** | 1.00 [Reference] | 1.17 (0.95-1.44) | 1.06 (0.86-1.30) | 1.35 (1.11-1.64) |  | 1.00 [Reference] | 0.89 (0.73-1.08) | 1.13 (0.92-1.38) | 1.14 (0.97-1.33) |
| ***P* value** |  | 0.141 | 0.588 | 0.003 |  |  | 0.245 | 0.242 | 0.107 |
| **Model 3 HR (95% CI) ^c^** | 1.00 [Reference] | 1.24 (1.01-1.54) | 1.12 (0.91-1.37) | 1.46 (1.19-1.78) |  | 1.00 [Reference] | 0.91 (0.75-1.11) | 1.14 (0.93-1.39) | 1.17 (1.00-1.37) |
| ***P* value** |  | 0.045 | 0.299 | <0.001 |  |  | 0.364 | 0.197 | 0.057 |
| **Model 4 HR (95% CI) ^d^** | 1.00 [Reference] | 1.29 (0.99-1.69) | 1.09 (0.88-1.34) | 1.49 (1.15-1.94) |  | 1.00 [Reference] | 1.09 (0.83-1.42) | 1.13 (0.93-1.38) | 1.40 (1.09-1.79) |
| ***P* value** |  | 0.062 | 0.434 | 0.003 |  |  | 0.549 | 0.221 | 0.008 |
| ***P* _interaction_ ^e^** |  |  |  | 0.091 |  |  |  |  | 0.732 |
| **All-cause mortality** |  |  |  |  |  |  |  |  |  |
| **Men** |  |  |  |  |  |  |  |  |  |
| **N** | 7018 | 573 | 640 | 661 |  | 7408 | 466 | 420 | 598 |
| **Cases/Person-Years** | 398/57,484 | 41/4668 | 45/5198 | 58/5379 |  | 433/60,632 | 35/3809 | 29/3427 | 45/4861 |
| **Model 1 HR (95% CI) ^a^** | 1.00 [Reference] | 1.39 (1.01-1.92) | 1.44 (1.05-1.95) | 1.80 (1.36-2.37) |  | 1.00 [Reference] | 1.54 (1.09-2.17) | 1.48 (1.02-2.16) | 1.60 (1.17-2.17) |
| ***P* value** |  | 0.043 | 0.022 | <0.001 |  |  | 0.014 | 0.041 | 0.003 |
| **Model 2 HR (95% CI) ^b^** | 1.00 [Reference] | 1.34 (0.97-1.85) | 1.28 (0.94-1.75) | 1.57 (1.18-2.08) |  | 1.00 [Reference] | 1.31 (0.92-1.85) | 1.34 (0.92-1.96) | 1.30 (0.95-1.77) |
| ***P* value** |  | 0.081 | 0.115 | 0.002 |  |  | 0.131 | 0.128 | 0.102 |
| **Model 3 HR (95% CI) ^c^** | 1.00 [Reference] | 1.18 (0.84-1.64) | 1.19 (0.87-1.63) | 1.30 (0.96-1.76) |  | 1.00 [Reference] | 1.16 (0.82-1.66) | 1.28 (0.88-1.87) | 1.13 (0.82-1.55) |
| ***P* value** |  | 0.341 | 0.286 | 0.092 |  |  | 0.397 | 0.199 | 0.468 |
| **Model 4 HR (95% CI) ^d^** | 1.00 [Reference] | 1.06 (0.69-1.65) | 1.21 (0.88-1.67) | 1.23 (0.80-1.88) |  | 1.00 [Reference] | 1.23 (0.81-1.87) | 1.33 (0.91-1.95) | 1.22 (0.81-1.82) |
| ***P* value** |  | 0.782 | 0.236 | 0.351 |  |  | 0.330 | 0.137 | 0.344 |
| **Women** |  |  |  |  |  |  |  |  |  |
| **N** | 7783 | 501 | 579 | 503 |  | 7213 | 670 | 546 | 937 |
| **Cases/Person-Years** | 260/64,311 | 17/4125 | 16/4791 | 21/4132 |  | 233/59,631 | 21/5520 | 21/4489 | 39/7720 |
| **Model 1 HR (95% CI) ^a^** | 1.00 [Reference] | 1.16 (0.71-1.89) | 0.90 (0.55-1.50) | 1.43 (0.91-2.23) |  | 1.00 [Reference] | 1.08 (0.69-1.69) | 1.34 (0.86-2.10) | 1.45 (1.03-2.04) |
| ***P* value** |  | 0.559 | 0.696 | 0.117 |  |  | 0.741 | 0.194 | 0.032 |
| **Model 2 HR (95% CI) ^b^** | 1.00 [Reference] | 1.04 (0.63-1.71) | 0.81 (0.49-1.34) | 1.21 (0.77-1.91) |  | 1.00 [Reference] | 0.94 (0.60-1.48) | 1.22 (0.78-1.91) | 1.28 (0.91-1.80) |
| ***P* value** |  | 0.873 | 0.406 | 0.404 |  |  | 0.791 | 0.380 | 0.160 |
| **Model 3 HR (95% CI) ^c^** | 1.00 [Reference] | 1.06 (0.64-1.77) | 0.82 (0.49-1.37) | 1.25 (0.78-2.00) |  | 1.00 [Reference] | 0.95 (0.61-1.50) | 1.23 (0.79-1.93) | 1.30 (0.92-1.84) |
| ***P* value** |  | 0.808 | 0.453 | 0.361 |  |  | 0.840 | 0.366 | 0.143 |
| **Model 4 HR (95% CI) ^d^** | 1.00 [Reference] | 0.95 (0.51-1.76) | 0.79 (0.47-1.32) | 1.10 (0.60-2.01) |  | 1.00 [Reference] | 0.88 (0.50-1.58) | 1.22 (0.78-1.91) | 1.20 (0.71-2.02) |
| ***P* value** |  | 0.863 | 0.361 | 0.765 |  |  | 0.679 | 0.383 | 0.497 |
| ***P* _interaction_ ^e^** |  |  |  | 0.108 |  |  |  |  | 0.694 |
| **CVD mortality** |  |  |  |  |  |  |  |  |  |
| **Men** |  |  |  |  |  |  |  |  |  |
| **N** | 7018 | 573 | 640 | 661 |  | 7408 | 466 | 420 | 598 |
| **Cases/Person-Years** | 151/58,323 | 18/4740 | 17/5296 | 28/5464 |  | 161/61,549 | 15/3860 | 15/3471 | 23/4944 |
| **Model 1 HR (95% CI) ^a^** | 1.00 [Reference] | 1.55 (0.95-2.53) | 1.41 (0.85-2.32) | 2.08 (1.37-3.15) |  | 1.00 [Reference] | 1.80 (1.06-3.06) | 2.08 (1.22-3.53) | 2.22 (1.43-3.44) |
| ***P* value** |  | 0.081 | 0.182 | 0.001 |  |  | 0.030 | 0.007 | <0.001 |
| **Model 2 HR (95% CI) ^b^** | 1.00 [Reference] | 1.47 (0.89-2.41) | 1.21 (0.73-2.00) | 1.70 (1.10-2.62) |  | 1.00 [Reference] | 1.44 (0.84-2.46) | 1.83 (1.07-3.12) | 1.63 (1.04-2.55) |
| ***P* value** |  | 0.129 | 0.462 | 0.016 |  |  | 0.180 | 0.027 | 0.032 |
| **Model 3 HR (95% CI) ^c^** | 1.00 [Reference] | 1.21 (0.73-2.03) | 1.07 (0.64-1.79) | 1.29 (0.81-2.05) |  | 1.00 [Reference] | 1.23 (0.72-2.12) | 1.73 (1.01-2.95) | 1.35 (0.85-2.14) |
| ***P* value** |  | 0.455 | 0.786 | 0.285 |  |  | 0.449 | 0.045 | 0.202 |
| **Model 4 HR (95% CI) ^d^** | 1.00 [Reference] | 1.10 (0.56-2.17) | 1.13 (0.67-1.89) | 1.24 (0.64-2.42) |  | 1.00 [Reference] | 1.23 (0.64-2.33) | 1.80 (1.05-3.08) | 1.36 (0.75-2.48) |
| ***P* value** |  | 0.781 | 0.654 | 0.530 |  |  | 0.535 | 0.031 | 0.316 |
| **Women** |  |  |  |  |  |  |  |  |  |
| **N** | 7783 | 501 | 579 | 503 |  | 7213 | 670 | 546 | 937 |
| **Cases/Person-Years** | 79/64,832 | 6/4167 | 4/4827 | 7/4186 |  | 64/60,131 | 8/5561 | 11/4527 | 13/7794 |
| **Model 1 HR (95% CI) ^a^** | 1.00 [Reference] | 1.28 (0.56-2.97) | 0.75 (0.27-2.04) | 1.57 (0.72-3.41) |  | 1.00 [Reference] | 1.46 (0.69-3.05) | 2.58 (1.36-4.90) | 1.77 (0.97-3.21) |
| ***P* value** |  | 0.558 | 0.566 | 0.252 |  |  | 0.320 | 0.004 | 0.062 |
| **Model 2 HR (95% CI) ^b^** | 1.00 [Reference] | 1.14 (0.49-2.65) | 0.66 (0.24-1.81) | 1.31 (0.59-2.90) |  | 1.00 [Reference] | 1.17 (0.55-2.46) | 2.17 (1.14-4.13) | 1.45 (0.79-2.67) |
| ***P* value** |  | 0.760 | 0.418 | 0.512 |  |  | 0.686 | 0.019 | 0.225 |
| **Model 3 HR (95% CI) ^c^** | 1.00 [Reference] | 1.18 (0.50-2.81) | 0.68 (0.24-1.88) | 1.36 (0.59-3.12) |  | 1.00 [Reference] | 1.20 (0.56-2.53) | 2.19 (1.15-4.18) | 1.49 (0.81-2.76) |
| ***P* value** |  | 0.706 | 0.457 | 0.468 |  |  | 0.641 | 0.017 | 0.201 |
| **Model 4 HR (95% CI) ^d^** | 1.00 [Reference] | 1.01 (0.34-2.96) | 0.64 (0.23-1.78) | 1.14 (0.39-3.35) |  | 1.00 [Reference] | 1.16 (0.43-3.12) | 2.17 (1.14-4.13) | 1.44 (0.58-3.58) |
| ***P* value** |  | 0.989 | 0.388 | 0.808 |  |  | 0.773 | 0.019 | 0.429 |
| ***P* _interaction_ ^e^** |  |  |  | 0.286 |  |  |  |  | 0.875 |

^a^Model 1 adjusted for age and sex;

^b^Model 2 adjusted for age, sex, ethnicity, current employment status, education level, Townsend deprivation index, smoking status, alcohol consumption frequency, physical activity, TV watching time, healthy diet score, and longstanding illnesses, disability, or infirmity.

^c^Model 3 adjusted for age, sex, ethnicity, current employment status, education level, Townsend deprivation index, smoking status, alcohol consumption frequency, physical activity, TV watching time, healthy diet score, longstanding illnesses, disability, or infirmity, and marital status.

^d^Model 4 adjusted for age, sex, ethnicity, current employment status, education level, Townsend deprivation index, smoking status, alcohol consumption frequency, physical activity, TV watching time, healthy diet score, longstanding illnesses, disability, or infirmity, and social isolation or loneliness.

^e^*P* _interaction_ was calculated after adjusting for age*social isolation change or loneliness change, sex, ethnicity, current employment status, education level, Townsend deprivation index, smoking status, alcohol consumption frequency, physical activity, TV watching time, healthy diet score, and longstanding illnesses, disability, or infirmity.

CI=confidence interval; CVD=cardiovascular disease; HR=hazard ratio.

**Table S13. Associations of patterns of change in social isolation and loneliness and their cumulative score with cardiac function by CMR among participants free of CVD additionally adjusting marital status**

|  | **Transient** | |  | **Incident** | |  | **Persistent** | |  | **Cumulative score** | |
| --- | --- | --- | --- | --- | --- | --- | --- | --- | --- | --- | --- |
|  | **β (95% CI) ^a^** | ***P* value** |  | **β (95% CI) ^a^** | ***P* value** |  | **β (95% CI) ^a^** | ***P* value** |  | **β (95% CI) ^a^** | ***P* value** |
|  |  |  |  |  |  |  |  |  |  |  |  |
| **Social isolation** |  |  |  |  |  |  |  |  |  |  |  |
| Cardiac index | 0.01 (-0.05, 0.07) | 0.634 |  | 0.00 (-0.05, 0.06) | 0.866 |  | 0.02 (-0.04, 0.07) | 0.513 |  | -0.00 (-0.01, 0.01) | 0.893 |
| Cardiac output | -0.01 (-0.12, 0.11) | 0.902 |  | -0.02 (-0.12, 0.08) | 0.690 |  | 0.01 (-0.11, 0.13) | 0.852 |  | -0.01 (-0.03, 0.01) | 0.292 |
| LVEF | -0.00 (-0.70, 0.70) | 0.995 |  | -0.26 (-0.91, 0.39) | 0.428 |  | -0.32 (-0.99, 0.34) | 0.338 |  | -0.04 (-0.14, 0.06) | 0.396 |
| LVSV | -1.41 (-3.28, 0.46) | 0.139 |  | -1.38 (-3.02, 0.27) | 0.102 |  | -1.97 (-3.88, -0.07) | 0.042 |  | -0.49 (-0.77, -0.22) | <0.001 |
| **Loneliness** |  |  |  |  |  |  |  |  |  |  |  |
| Cardiac index | -0.03 (-0.09, 0.02) | 0.227 |  | 0.02 (-0.03, 0.08) | 0.430 |  | -0.09 (-0.14, -0.04) | <0.001 |  | -0.03 (-0.04, -0.01) | 0.001 |
| Cardiac output | -0.07 (-0.18, 0.03) | 0.172 |  | 0.04 (-0.08, 0.16) | 0.517 |  | -0.15 (-0.24, -0.05) | 0.002 |  | -0.04 (-0.07, -0.01) | 0.021 |
| LVEF | 0.09 (-0.51, 0.69) | 0.759 |  | 0.57 (-0.12, 1.27) | 0.104 |  | -0.29 (-0.87, 0.28) | 0.319 |  | -0.06 (-0.25, 0.13) | 0.534 |
| LVSV | -0.13 (-1.95, 1.68) | 0.884 |  | -0.48 (-2.35, 1.38) | 0.612 |  | -1.97 (-3.59, -0.34) | 0.018 |  | -0.46 (-0.99, 0.08) | 0.094 |

^a^β were adjusted for age, sex, ethnicity, marital status, current employment status, education level, Townsend deprivation index, smoking status, alcohol consumption frequency, physical activity, TV watching time, healthy diet score, and longstanding illnesses, disability, or infirmity. The references here are participants who were never socially isolated or never lonely.

CMR=cardiac magnetic resonance; CVD=cardiovascular disease; LVEF=cardiovascular disease; LVSV=left ventricular stroke volume.

**Table S14. Associations of patterns of change in social isolation and loneliness and their cumulative score with cardiac function by CMR among participants free of CVD mutually adjusting social isolation score or loneliness score at baseline**

|  | **Transient** | |  | **Incident** | |  | **Persistent** | |  | **Cumulative score** | |
| --- | --- | --- | --- | --- | --- | --- | --- | --- | --- | --- | --- |
|  | **β (95% CI) ^a^** | ***P* value** |  | **β (95% CI) ^a^** | ***P* value** |  | **β (95% CI) ^a^** | ***P* value** |  | **β (95% CI) ^a^** | ***P* value** |
| **Social isolation** |  |  |  |  |  |  |  |  |  |  |  |
| Cardiac index | 0.02 (-0.04, 0.07) | 0.608 |  | 0.00 (-0.05, 0.05) | 0.967 |  | 0.02 (-0.04, 0.07) | 0.533 |  | -0.00 (-0.01, 0.01) | 0.837 |
| Cardiac output | -0.00 (-0.12, 0.11) | 0.963 |  | -0.02 (-0.12, 0.08) | 0.694 |  | 0.02 (-0.10, 0.13) | 0.784 |  | -0.01 (-0.02, 0.01) | 0.397 |
| LVEF | -0.04 (-0.73, 0.64) | 0.898 |  | -0.31 (-0.95, 0.33) | 0.344 |  | -0.40 (-1.05, 0.25) | 0.231 |  | -0.06 (-0.15, 0.03) | 0.197 |
| LVSV | -1.47 (-3.31, 0.37) | 0.118 |  | -1.44 (-3.08, 0.20) | 0.085 |  | -2.09 (-3.98, -0.20) | 0.030 |  | -0.48 (-0.73, -0.23) | <0.001 |
| **Loneliness** |  |  |  |  |  |  |  |  |  |  |  |
| Cardiac index | -0.04 (-0.09, 0.02) | 0.186 |  | 0.02 (-0.04, 0.08) | 0.449 |  | -0.09 (-0.14, -0.05) | <0.001 |  | -0.03 (-0.04, -0.01) | 0.001 |
| Cardiac output | -0.07 (-0.18, 0.03) | 0.172 |  | 0.04 (-0.08, 0.17) | 0.494 |  | -0.14 (-0.24, -0.05) | 0.003 |  | -0.04 (-0.07, -0.01) | 0.022 |
| LVEF | 0.05 (-0.55, 0.65) | 0.871 |  | 0.58 (-0.11, 1.26) | 0.101 |  | -0.33 (-0.91, 0.25) | 0.26 |  | -0.07 (-0.25, 0.12) | 0.478 |
| LVSV | -0.16 (-1.98, 1.65) | 0.861 |  | -0.42 (-2.26, 1.42) | 0.653 |  | -1.99 (-3.61, -0.37) | 0.016 |  | -0.45 (-0.98, 0.07) | 0.093 |

^a^ *β* were adjusted for age, sex, ethnicity, marital status, current employment status, education level, Townsend deprivation index, smoking status, alcohol consumption frequency, physical activity, TV watching time, healthy diet score, and longstanding illnesses, disability, or infirmity. The references here are participants who were never socially isolated or never lonely.

CMR=cardiac magnetic resonance; CVD=cardiovascular disease; LVEF=cardiovascular disease; LVSV=left ventricular stroke volume.

**Table S15. Associations of patterns of change in social isolation and loneliness with subsequent cardiac function by CMR in sample with complete cases**

|  | **Transient** | |  | **Incident** | |  | **Persistent** | |
| --- | --- | --- | --- | --- | --- | --- | --- | --- |
|  | ***β* (95% CI) ^a^** | ***P* value** |  | ***β* (95% CI) ^a^** | ***P* value** |  | ***β* (95% CI) ^a^** | ***P* value** |
| **Social isolation** |  |  |  |  |  |  |  |  |
| Cardiac index | -0.00 (-0.06, 0.06) | 0.991 |  | 0.00 (-0.05, 0.06) | 0.872 |  | 0.02 (-0.03, 0.08) | 0.414 |
| Cardiac output | -0.01 (-0.13, 0.11) | 0.828 |  | -0.01 (-0.12, 0.11) | 0.921 |  | 0.05 (-0.08, 0.17) | 0.462 |
| LVEF | -0.14 (-0.86, 0.58) | 0.707 |  | -0.35 (-1.04, 0.34) | 0.321 |  | -0.51 (-1.23, 0.21) | 0.162 |
| LVEDV | -2.67 (-5.94, 0.60) | 0.109 |  | -0.79 (-3.59, 2.01) | 0.580 |  | -1.42 (-4.89, 2.05) | 0.422 |
| LVESV | -0.36 (-2.21, 1.49) | 0.702 |  | 0.45 (-1.16, 2.05) | 0.586 |  | 0.65 (-1.30, 2.60) | 0.515 |
| LVSV | -1.93 (-3.92, 0.06) | 0.057 |  | -0.83 (-2.64, 0.99) | 0.372 |  | -1.80 (-3.82, 0.21) | 0.079 |
| **Loneliness** |  |  |  |  |  |  |  |  |
| Cardiac index | -0.04 (-0.10, 0.02) | 0.234 |  | 0.04 (-0.02, 0.10) | 0.217 |  | -0.09 (-0.14, -0.04) | <0.001 |
| Cardiac output | -0.06 (-0.17, 0.05) | 0.309 |  | 0.06 (-0.07, 0.20) | 0.353 |  | -0.14 (-0.24, -0.03) | 0.009 |
| LVEF | -0.01 (-0.64, 0.62) | 0.975 |  | 0.37 (-0.36, 1.09) | 0.318 |  | -0.43 (-1.04, 0.19) | 0.175 |
| LVEDV | -0.48 (-3.59, 2.62) | 0.761 |  | -1.14 (-4.68, 2.40) | 0.527 |  | -1.82 (-4.79, 1.15) | 0.230 |
| LVESV | -0.01 (-1.67, 1.64) | 0.989 |  | -0.63 (-2.68, 1.43) | 0.550 |  | -0.15 (-1.73, 1.44) | 0.856 |
| LVSV | -0.35 (-2.32, 1.63) | 0.731 |  | -0.30 (-2.22, 1.63) | 0.764 |  | -2.09 (-3.82, -0.37) | 0.017 |

^a^ *β* were adjusted for age, sex, ethnicity, current employment status, education level, Townsend deprivation index, smoking status, alcohol consumption frequency, physical activity, TV watching time, healthy diet score, and longstanding illnesses, disability, or infirmity. The references here are participants who were never socially isolated or never lonely.

CI=confidence interval; CMR=cardiac magnetic resonance; CVD=cardiovascular disease; LVEDV=left ventricular end diastolic volume; LVEF=left ventricular ejection fraction; LVESV=left ventricular end systolic volume; LVSV=left ventricular stroke volume.

**Table S16. Associations of patterns of change in social isolation and loneliness with subsequent cardiac function by CMR in sample with complete cases additionally adjusting marital status**

|  | **Transient** | |  | **Incident** | |  | **Persistent** | |
| --- | --- | --- | --- | --- | --- | --- | --- | --- |
|  | ***β* (95% CI) ^a^** | ***P* value** |  | ***β* (95% CI) ^a^** | ***P* value** |  | ***β* (95% CI) ^a^** | ***P* value** |
| **Social isolation** |  |  |  |  |  |  |  |  |
| Cardiac index | 0.01 (-0.06, 0.07) | 0.839 |  | 0.01 (-0.04, 0.07) | 0.691 |  | 0.04 (-0.02, 0.10) | 0.235 |
| Cardiac output | -0.01 (-0.13, 0.12) | 0.919 |  | 0.00 (-0.11, 0.11) | 0.987 |  | 0.06 (-0.07, 0.18) | 0.373 |
| LVEF | -0.09 (-0.82, 0.65) | 0.816 |  | -0.30 (-1.00, 0.39) | 0.393 |  | -0.43 (-1.15, 0.30) | 0.250 |
| LVEDV | -2.62 (-5.91, 0.68) | 0.119 |  | -0.74 (-3.59, 2.11) | 0.612 |  | -1.34 (-4.83, 2.16) | 0.454 |
| LVESV | -0.39 (-2.26, 1.48) | 0.686 |  | 0.42 (-1.21, 2.05) | 0.613 |  | 0.60 (-1.40, 2.61) | 0.556 |
| LVSV | -1.79 (-3.81, 0.23) | 0.083 |  | -0.70 (-2.51, 1.11) | 0.448 |  | -1.58 (-3.63, 0.46) | 0.129 |
| **Loneliness** |  |  |  |  |  |  |  |  |
| Cardiac index | -0.03 (-0.09, 0.03) | 0.272 |  | 0.04 (-0.02, 0.10) | 0.213 |  | -0.09 (-0.14, -0.04) | <0.001 |
| Cardiac output | -0.06 (-0.17, 0.06) | 0.322 |  | 0.06 (-0.07, 0.20) | 0.352 |  | -0.14 (-0.24, -0.03) | 0.011 |
| LVEF | 0.05 (-0.58, 0.69) | 0.871 |  | 0.40 (-0.33, 1.12) | 0.284 |  | -0.36 (-0.97, 0.26) | 0.261 |
| LVEDV | -0.40 (-3.54, 2.73) | 0.801 |  | -1.11 (-4.66, 2.44) | 0.541 |  | -1.72 (-4.73, 1.28) | 0.260 |
| LVESV | -0.06 (-1.74, 1.61) | 0.940 |  | -0.65 (-2.71, 1.41) | 0.535 |  | -0.21 (-1.82, 1.40) | 0.800 |
| LVSV | -0.20 (-2.19, 1.80) | 0.846 |  | -0.24 (-2.20, 1.71) | 0.807 |  | -1.90 (-3.66, -0.14) | 0.035 |

^a^ *β* were adjusted for age, sex, ethnicity, current employment status, education level, Townsend deprivation index, smoking status, alcohol consumption frequency, physical activity, TV watching time, healthy diet score, longstanding illnesses, disability, or infirmity, and marital status. The references here are participants who were never socially isolated or never lonely.

CI=confidence interval; CMR=cardiac magnetic resonance; CVD=cardiovascular disease; LVEDV=left ventricular end diastolic volume; LVEF=left ventricular ejection fraction; LVESV=left ventricular end systolic volume; LVSV=left ventricular stroke volume.

**Table S17. Associations of patterns of change in social isolation and loneliness with subsequent cardiac function by CMR in sample with complete cases mutually adjusting social isolation score or loneliness score at baseline**

|  | **Transient** | |  | **Incident** | |  | **Persistent** | |
| --- | --- | --- | --- | --- | --- | --- | --- | --- |
|  | ***β* (95% CI) ^a^** | ***P* value** |  | ***β* (95% CI) ^a^** | ***P* value** |  | ***β* (95% CI) ^a^** | ***P* value** |
| **Social isolation** |  |  |  |  |  |  |  |  |
| Cardiac index | 0.01 (-0.06, 0.07) | 0.816 |  | 0.01 (-0.05, 0.06) | 0.760 |  | 0.04 (-0.02, 0.10) | 0.230 |
| Cardiac output | -0.00 (-0.13, 0.12) | 0.944 |  | 0.00 (-0.11, 0.11) | 0.995 |  | 0.06 (-0.06, 0.19) | 0.340 |
| LVEF | -0.11 (-0.83, 0.60) | 0.754 |  | -0.34 (-1.03, 0.35) | 0.331 |  | -0.48 (-1.19, 0.24) | 0.192 |
| LVEDV | -2.64 (-5.92, 0.63) | 0.114 |  | -0.77 (-3.57, 2.03) | 0.591 |  | -1.38 (-4.84, 2.08) | 0.434 |
| LVESV | -0.38 (-2.24, 1.47) | 0.686 |  | 0.43 (-1.18, 2.03) | 0.601 |  | 0.61 (-1.34, 2.57) | 0.537 |
| LVSV | -1.87 (-3.87, 0.13) | 0.067 |  | -0.78 (-2.59, 1.03) | 0.399 |  | -1.71 (-3.75, 0.33) | 0.100 |
| **Loneliness** |  |  |  |  |  |  |  |  |
| Cardiac index | -0.04 (-0.10, 0.02) | 0.234 |  | 0.04 (-0.02, 0.10) | 0.221 |  | -0.09 (-0.15, -0.04) | <0.001 |
| Cardiac output | -0.06 (-0.17, 0.06) | 0.324 |  | 0.07 (-0.07, 0.20) | 0.338 |  | -0.13 (-0.24, -0.03) | 0.011 |
| LVEF | 0.03 (-0.60, 0.65) | 0.935 |  | 0.41 (-0.32, 1.14) | 0.268 |  | -0.37 (-0.99, 0.25) | 0.239 |
| LVEDV | -0.42 (-3.51, 2.67) | 0.791 |  | -1.06 (-4.59, 2.48) | 0.558 |  | -1.71 (-4.68, 1.25) | 0.257 |
| LVESV | -0.07 (-1.73, 1.60) | 0.936 |  | -0.69 (-2.75, 1.37) | 0.513 |  | -0.22 (-1.82, 1.38) | 0.787 |
| LVSV | -0.23 (-2.22, 1.76) | 0.823 |  | -0.16 (-2.13, 1.80) | 0.872 |  | -1.93 (-3.68, -0.18) | 0.031 |

^a^ *β* were adjusted for age, sex, ethnicity, marital status, current employment status, education level, Townsend deprivation index, smoking status, alcohol consumption frequency, physical activity, TV watching time, healthy diet score, longstanding illnesses, disability, or infirmity, and social isolation or loneliness. The references here are participants who were never socially isolated or never lonely.

CI=confidence interval; CMR=cardiac magnetic resonance; CVD=cardiovascular disease; LVEDV=left ventricular end diastolic volume; LVEF=left ventricular ejection fraction; LVESV=left ventricular end systolic volume; LVSV=left ventricular stroke volume.

**Table S18. Associations of patterns of change in social isolation and loneliness with subsequent cardiac function by CMR stratified by sex**

|  | **Transient** | |  | **Incident** | |  | **Persistent** | |
| --- | --- | --- | --- | --- | --- | --- | --- | --- |
|  | ***β* (95% CI) ^a^** | ***P* value** |  | ***β* (95% CI) ^a^** | ***P* value** |  | ***β* (95% CI) ^a^** | ***P* value** |
| **Men** |  |  |  |  |  |  |  |  |
| **Social isolation** |  |  |  |  |  |  |  |  |
| Cardiac index | 0.03 (-0.05, 0.11) | 0.425 |  | 0.05 (-0.03, 0.12) | 0.216 |  | 0.03 (-0.04, 0.11) | 0.379 |
| Cardiac output | 0.00 (-0.17, 0.17) | 0.985 |  | 0.02 (-0.14, 0.18) | 0.845 |  | 0.03 (-0.13, 0.19) | 0.720 |
| LVEF | 0.21 (-0.79, 1.20) | 0.685 |  | 0.08 (-0.86, 1.01) | 0.868 |  | -0.36 (-1.34, 0.63) | 0.477 |
| LVEDV | -4.25 (-9.35, 0.85) | 0.103 |  | -3.70 (-7.98, 0.58) | 0.090 |  | -6.58 (-11.02, -2.14) | 0.004 |
| LVESV | -1.00 (-4.03, 2.04) | 0.520 |  | -1.51 (-4.00, 0.98) | 0.235 |  | -1.82 (-4.44, 0.80) | 0.173 |
| LVSV | -1.95 (-4.81, 0.91) | 0.182 |  | -1.87 (-4.47, 0.74) | 0.161 |  | -4.63 (-7.42, -1.85) | 0.001 |
| **Loneliness** |  |  |  |  |  |  |  |  |
| Cardiac index | -0.04 (-0.13, 0.06) | 0.414 |  | 0.02 (-0.06, 0.11) | 0.581 |  | -0.06 (-0.13, 0.02) | 0.149 |
| Cardiac output | -0.10 (-0.29, 0.10) | 0.328 |  | 0.04 (-0.17, 0.24) | 0.733 |  | -0.13 (-0.29, 0.03) | 0.106 |
| LVEF | 0.31 (-0.73, 1.34) | 0.564 |  | -0.45 (-1.48, 0.59) | 0.398 |  | -0.21 (-1.20, 0.78) | 0.678 |
| LVEDV | -6.96 (-12.36, -1.56) | 0.012 |  | -2.36 (-7.97, 3.25) | 0.409 |  | -4.86 (-10.10, 0.38) | 0.069 |
| LVESV | -3.24 (-6.06, -0.42) | 0.024 |  | 0.10 (-3.25, 3.45) | 0.953 |  | -1.45 (-4.45, 1.54) | 0.340 |
| LVSV | -3.44 (-7.09, 0.20) | 0.064 |  | -1.43 (-4.87, 2.02) | 0.417 |  | -2.85 (-5.71, 0.02) | 0.051 |
| **Women** |  |  |  |  |  |  |  |  |
| **Social isolation** |  |  |  |  |  |  |  |  |
| Cardiac index | -0.02 (-0.11, 0.07) | 0.669 |  | -0.05 (-0.12, 0.02) | 0.173 |  | -0.02 (-0.10, 0.06) | 0.643 |
| Cardiac output | -0.04 (-0.18, 0.11) | 0.637 |  | -0.07 (-0.20, 0.06) | 0.298 |  | -0.02 (-0.19, 0.14) | 0.774 |
| LVEF | -0.30 (-1.28, 0.68) | 0.545 |  | -0.70 (-1.58, 0.17) | 0.114 |  | -0.51 (-1.38, 0.36) | 0.248 |
| LVEDV | -0.55 (-4.53, 3.43) | 0.787 |  | -0.88 (-3.82, 2.06) | 0.555 |  | 1.75 (-2.62, 6.12) | 0.433 |
| LVESV | -0.06 (-2.24, 2.12) | 0.959 |  | 0.49 (-1.19, 2.18) | 0.568 |  | 1.41 (-0.95, 3.77) | 0.240 |
| LVSV | -1.22 (-3.72, 1.28) | 0.340 |  | -1.21 (-3.24, 0.81) | 0.239 |  | 0.38 (-2.08, 2.85) | 0.761 |
| **Loneliness** |  |  |  |  |  |  |  |  |
| Cardiac index | -0.03 (-0.10, 0.03) | 0.306 |  | 0.02 (-0.06, 0.10) | 0.617 |  | -0.12 (-0.18, -0.06) | <0.001 |
| Cardiac output | -0.06 (-0.18, 0.06) | 0.346 |  | 0.04 (-0.11, 0.20) | 0.566 |  | -0.16 (-0.28, -0.05) | 0.005 |
| LVEF | -0.17 (-0.90, 0.56) | 0.653 |  | 1.29 (0.43, 2.15) | 0.003 |  | -0.48 (-1.18, 0.23) | 0.185 |
| LVEDV | 2.88 (-0.31, 6.07) | 0.077 |  | -1.68 (-5.67, 2.32) | 0.411 |  | -0.96 (-3.99, 2.07) | 0.534 |
| LVESV | 1.58 (-0.17, 3.33) | 0.077 |  | -1.90 (-4.13, 0.33) | 0.095 |  | 0.24 (-1.29, 1.77) | 0.758 |
| LVSV | 1.29 (-0.81, 3.39) | 0.227 |  | 0.01 (-2.21, 2.23) | 0.993 |  | -1.82 (-3.71, 0.06) | 0.058 |

^a^ *β* were adjusted for age, sex, ethnicity, current employment status, education level, Townsend deprivation index, smoking status, alcohol consumption frequency, physical activity, TV watching time, healthy diet score, and longstanding illnesses, disability, or infirmity.

CI=confidence interval; CMR=cardiac magnetic resonance; CVD=cardiovascular disease; LVEDV=left ventricular end diastolic volume; LVEF=left ventricular ejection fraction; LVESV=left ventricular end systolic volume; LVSV=left ventricular stroke volume.

The references here are participants who were never socially isolated or never lonely.

**Table S19. Associations of patterns of change in social isolation and loneliness with subsequent cardiac function by CMR stratified by sex additionally adjusting marital status**

|  | **Transient** | |  | **Incident** | |  | **Persistent** | |
| --- | --- | --- | --- | --- | --- | --- | --- | --- |
|  | ***β* (95% CI) ^a^** | ***P* value** |  | ***β* (95% CI) ^a^** | ***P* value** |  | ***β* (95% CI) ^a^** | ***P* value** |
| **Men** |  |  |  |  |  |  |  |  |
| **Social isolation** |  |  |  |  |  |  |  |  |
| Cardiac index | 0.04 (-0.05, 0.12) | 0.380 |  | 0.05 (-0.02, 0.12) | 0.197 |  | 0.04 (-0.04, 0.12) | 0.313 |
| Cardiac output | 0.01 (-0.16, 0.18) | 0.904 |  | 0.02 (-0.14, 0.18) | 0.786 |  | 0.04 (-0.12, 0.20) | 0.597 |
| LVEF | 0.16 (-0.85, 1.17) | 0.753 |  | 0.05 (-0.88, 0.98) | 0.915 |  | -0.43 (-1.41, 0.55) | 0.389 |
| LVEDV | -3.72 (-8.85, 1.40) | 0.154 |  | -3.37 (-7.77, 1.02) | 0.132 |  | -5.68 (-10.27, -1.08) | 0.016 |
| LVESV | -0.68 (-3.75, 2.40) | 0.667 |  | -1.27 (-3.80, 1.26) | 0.325 |  | -1.23 (-3.99, 1.52) | 0.381 |
| LVSV | -1.69 (-4.60, 1.22) | 0.255 |  | -1.71 (-4.42, 0.99) | 0.214 |  | -4.16 (-7.02, -1.31) | 0.004 |
| **Loneliness** |  |  |  |  |  |  |  |  |
| Cardiac index | -0.04 (-0.14, 0.05) | 0.400 |  | 0.02 (-0.06, 0.11) | 0.586 |  | -0.06 (-0.14, 0.02) | 0.143 |
| Cardiac output | -0.09 (-0.29, 0.10) | 0.340 |  | 0.04 (-0.17, 0.24) | 0.731 |  | -0.13 (-0.29, 0.03) | 0.117 |
| LVEF | 0.28 (-0.75, 1.31) | 0.593 |  | -0.46 (-1.50, 0.58) | 0.385 |  | -0.25 (-1.25, 0.75) | 0.621 |
| LVEDV | -6.40 (-11.86, -0.95) | 0.021 |  | -2.23 (-8.03, 3.56) | 0.450 |  | -4.08 (-9.35, 1.19) | 0.129 |
| LVESV | -2.97 (-5.86, -0.07) | 0.045 |  | 0.19 (-3.17, 3.54) | 0.914 |  | -1.02 (-4.10, 2.06) | 0.516 |
| LVSV | -3.14 (-6.74, 0.46) | 0.087 |  | -1.36 (-4.81, 2.09) | 0.440 |  | -2.39 (-5.31, 0.52) | 0.108 |
| **Women** |  |  |  |  |  |  |  |  |
| **Social isolation** |  |  |  |  |  |  |  |  |
| Cardiac index | -0.01 (-0.10, 0.08) | 0.825 |  | -0.04 (-0.11, 0.03) | 0.284 |  | -0.00 (-0.08, 0.07) | 0.902 |
| Cardiac output | -0.03 (-0.18, 0.12) | 0.686 |  | -0.06 (-0.20, 0.07) | 0.345 |  | -0.02 (-0.19, 0.15) | 0.835 |
| LVEF | -0.16 (-1.13, 0.81) | 0.751 |  | -0.56 (-1.45, 0.32) | 0.214 |  | -0.30 (-1.18, 0.58) | 0.503 |
| LVEDV | -0.81 (-4.78, 3.17) | 0.692 |  | -1.21 (-4.24, 1.82) | 0.435 |  | 1.33 (-3.07, 5.74) | 0.553 |
| LVESV | -0.32 (-2.53, 1.88) | 0.773 |  | 0.17 (-1.54, 1.87) | 0.849 |  | 0.94 (-1.47, 3.35) | 0.445 |
| LVSV | -1.17 (-3.69, 1.35) | 0.362 |  | -1.16 (-3.23, 0.90) | 0.269 |  | 0.45 (-2.06, 2.96) | 0.726 |
| **Loneliness** |  |  |  |  |  |  |  |  |
| Cardiac index | -0.03 (-0.09, 0.04) | 0.412 |  | 0.02 (-0.05, 0.10) | 0.573 |  | -0.12 (-0.17, -0.06) | <0.001 |
| Cardiac output | -0.06 (-0.18, 0.07) | 0.366 |  | 0.04 (-0.11, 0.20) | 0.561 |  | -0.16 (-0.28, -0.05) | 0.007 |
| LVEF | -0.01 (-0.75, 0.73) | 0.969 |  | 1.36 (0.50, 2.21) | 0.002 |  | -0.32 (-1.04, 0.39) | 0.377 |
| LVEDV | 2.64 (-0.62, 5.90) | 0.112 |  | -1.78 (-5.80, 2.24) | 0.385 |  | -1.20 (-4.25, 1.86) | 0.443 |
| LVESV | 1.28 (-0.43, 2.98) | 0.143 |  | -2.04 (-4.28, 0.19) | 0.073 |  | -0.05 (-1.60, 1.51) | 0.955 |
| LVSV | 1.33 (-0.76, 3.43) | 0.212 |  | 0.03 (-2.20, 2.25) | 0.981 |  | -1.77 (-3.69, 0.14) | 0.070 |

^a^ *β* were adjusted for age, sex, ethnicity, current employment status, education level, Townsend deprivation index, smoking status, alcohol consumption frequency, physical activity, TV watching time, healthy diet score, longstanding illnesses, disability, or infirmity, and marital status. The references here are participants who were never socially isolated or never lonely.

CI=confidence interval; CMR=cardiac magnetic resonance; CVD=cardiovascular disease; LVEDV=left ventricular end diastolic volume; LVEF=left ventricular ejection fraction; LVESV=left ventricular end systolic volume; LVSV=left ventricular stroke volume.

**Table S20. Associations of patterns of change in social isolation and loneliness with subsequent cardiac function by CMR stratified by sex mutually adjusting social isolation score or loneliness score at baseline**

|  | **Transient** | |  | **Incident** | |  | **Persistent** | |
| --- | --- | --- | --- | --- | --- | --- | --- | --- |
|  | ***β* (95% CI) ^a^** | ***P* value** |  | ***β* (95% CI) ^a^** | ***P* value** |  | ***β* (95% CI) ^a^** | ***P* value** |
| **Men** |  |  |  |  |  |  |  |  |
| **Social isolation** |  |  |  |  |  |  |  |  |
| Cardiac index | 0.04 (-0.04, 0.12) | 0.343 |  | 0.05 (-0.02, 0.12) | 0.179 |  | 0.04 (-0.03, 0.12) | 0.261 |
| Cardiac output | 0.01 (-0.16, 0.18) | 0.903 |  | 0.03 (-0.14, 0.19) | 0.758 |  | 0.05 (-0.11, 0.21) | 0.547 |
| LVEF | 0.24 (-0.75, 1.23) | 0.637 |  | 0.11 (-0.83, 1.05) | 0.821 |  | -0.29 (-1.26, 0.68) | 0.552 |
| LVEDV | -4.10 (-9.20, 1.01) | 0.116 |  | -3.56 (-7.94, 0.82) | 0.111 |  | -6.26 (-10.70, -1.82) | 0.006 |
| LVESV | -0.98 (-4.02, 2.06) | 0.528 |  | -1.49 (-3.99, 1.00) | 0.241 |  | -1.77 (-4.41, 0.86) | 0.186 |
| LVSV | -1.81 (-4.69, 1.07) | 0.217 |  | -1.75 (-4.43, 0.93) | 0.201 |  | -4.38 (-7.17, -1.59) | 0.002 |
| **Loneliness** |  |  |  |  |  |  |  |  |
| Cardiac index | -0.04 (-0.13, 0.06) | 0.429 |  | 0.03 (-0.06, 0.11) | 0.567 |  | -0.06 (-0.13, 0.02) | 0.161 |
| Cardiac output | -0.09 (-0.28, 0.10) | 0.369 |  | 0.04 (-0.17, 0.25) | 0.688 |  | -0.12 (-0.28, 0.04) | 0.132 |
| LVEF | 0.31 (-0.73, 1.34) | 0.564 |  | -0.45 (-1.49, 0.60) | 0.402 |  | -0.21 (-1.20, 0.78) | 0.678 |
| LVEDV | -6.55 (-12.00, -1.10) | 0.018 |  | -1.97 (-7.63, 3.68) | 0.494 |  | -4.38 (-9.63, 0.87) | 0.102 |
| LVESV | -3.13 (-6.03, -0.23) | 0.034 |  | 0.21 (-3.15, 3.57) | 0.903 |  | -1.32 (-4.30, 1.66) | 0.384 |
| LVSV | -3.15 (-6.89, 0.59) | 0.098 |  | -1.16 (-4.57, 2.25) | 0.505 |  | -2.55 (-5.44, 0.33) | 0.083 |
| **Women** |  |  |  |  |  |  |  |  |
| **Social isolation** |  |  |  |  |  |  |  |  |
| Cardiac index | -0.01 (-0.10, 0.08) | 0.870 |  | -0.05 (-0.11, 0.02) | 0.198 |  | -0.01 (-0.08, 0.07) | 0.858 |
| Cardiac output | -0.02 (-0.17, 0.12) | 0.761 |  | -0.06 (-0.19, 0.06) | 0.327 |  | -0.01 (-0.18, 0.16) | 0.896 |
| LVEF | -0.29 (-1.27, 0.69) | 0.559 |  | -0.70 (-1.58, 0.17) | 0.115 |  | -0.50 (-1.37, 0.37) | 0.258 |
| LVEDV | -0.71 (-4.69, 3.27) | 0.726 |  | -0.98 (-3.94, 1.98) | 0.516 |  | 1.58 (-2.82, 5.98) | 0.481 |
| LVESV | -0.17 (-2.37, 2.03) | 0.879 |  | 0.44 (-1.26, 2.14) | 0.612 |  | 1.31 (-1.06, 3.69) | 0.277 |
| LVSV | -1.25 (-3.76, 1.26) | 0.328 |  | -1.23 (-3.26, 0.80) | 0.236 |  | 0.35 (-2.13, 2.84) | 0.779 |
| **Loneliness** |  |  |  |  |  |  |  |  |
| Cardiac index | -0.03 (-0.10, 0.03) | 0.304 |  | 0.02 (-0.06, 0.10) | 0.625 |  | -0.12 (-0.18, -0.06) | <0.001 |
| Cardiac output | -0.06 (-0.18, 0.06) | 0.346 |  | 0.04 (-0.11, 0.20) | 0.567 |  | -0.17 (-0.28, -0.05) | 0.006 |
| LVEF | -0.11 (-0.84, 0.61) | 0.758 |  | 1.34 (0.48, 2.20) | 0.002 |  | -0.39 (-1.11, 0.33) | 0.286 |
| LVEDV | 2.81 (-0.40, 6.02) | 0.086 |  | -1.74 (-5.78, 2.29) | 0.397 |  | -1.08 (-4.11, 1.94) | 0.482 |
| LVESV | 1.48 (-0.24, 3.19) | 0.091 |  | -1.98 (-4.22, 0.25) | 0.082 |  | 0.09 (-1.46, 1.63) | 0.913 |
| LVSV | 1.30 (-0.78, 3.38) | 0.221 |  | 0.02 (-2.20, 2.25) | 0.985 |  | -1.80 (-3.71, 0.11) | 0.064 |

^a^ *β* were adjusted for age, sex, ethnicity, current employment status, education level, Townsend deprivation index, smoking status, alcohol consumption frequency, physical activity, TV watching time, healthy diet score, longstanding illnesses, disability, or infirmity, and social isolation or loneliness. The references here are participants who were never socially isolated or never lonely.

CI=confidence interval; CMR=cardiac magnetic resonance; CVD=cardiovascular disease; LVEDV=left ventricular end diastolic volume; LVEF=left ventricular ejection fraction; LVESV=left ventricular end systolic volume; LVSV=left ventricular stroke volume.

**Table S21. Associations of patterns of change in social isolation and loneliness with subsequent cardiac function by CMR stratified by age**

|  | **Transient** | |  | **Incident** | |  | **Persistent** | |
| --- | --- | --- | --- | --- | --- | --- | --- | --- |
|  | ***β* (95% CI) ^a^** | **P value** |  | ***β* (95% CI) ^a^** | **P value** |  | ***β* (95% CI) ^a^** | **P value** |
| **<60 years** |  |  |  |  |  |  |  |  |
| **Social isolation** |  |  |  |  |  |  |  |  |
| Cardiac index | -0.02 (-0.09, 0.06) | 0.678 |  | 0.02 (-0.03, 0.08) | 0.421 |  | -0.00 (-0.06, 0.06) | 0.994 |
| Cardiac output | -0.05 (-0.19, 0.08) | 0.461 |  | 0.01 (-0.12, 0.13) | 0.898 |  | -0.02 (-0.15, 0.11) | 0.732 |
| LVEF | -0.04 (-0.84, 0.76) | 0.922 |  | -0.26 (-0.97, 0.44) | 0.466 |  | -0.73 (-1.40, -0.06) | 0.032 |
| LVEDV | -1.87 (-5.58, 1.85) | 0.325 |  | -0.67 (-3.66, 2.33) | 0.663 |  | -2.01 (-5.75, 1.73) | 0.293 |
| LVESV | -0.30 (-2.38, 1.78) | 0.777 |  | 0.37 (-1.34, 2.08) | 0.671 |  | 0.57 (-1.49, 2.63) | 0.586 |
| LVSV | -1.68 (-3.79, 0.43) | 0.119 |  | -0.68 (-2.59, 1.23) | 0.486 |  | -2.43 (-4.57, -0.30) | 0.026 |
| **Loneliness** |  |  |  |  |  |  |  |  |
| Cardiac index | -0.06 (-0.12, 0.00) | 0.054 |  | 0.03 (-0.04, 0.09) | 0.446 |  | -0.10 (-0.16, -0.05) | <0.001 |
| Cardiac output | -0.12 (-0.24, 0.00) | 0.051 |  | 0.06 (-0.08, 0.19) | 0.425 |  | -0.18 (-0.29, -0.07) | 0.002 |
| LVEF | -0.15 (-0.78, 0.48) | 0.645 |  | 0.63 (-0.11, 1.38) | 0.096 |  | -0.69 (-1.33, -0.05) | 0.034 |
| LVEDV | -1.57 (-4.99, 1.84) | 0.367 |  | -1.36 (-5.16, 2.44) | 0.483 |  | -2.58 (-5.86, 0.71) | 0.124 |
| LVESV | -0.29 (-2.11, 1.52) | 0.750 |  | -1.06 (-3.18, 1.06) | 0.326 |  | 0.01 (-1.63, 1.66) | 0.989 |
| LVSV | -1.29 (-3.44, 0.87) | 0.242 |  | 0.01 (-2.06, 2.08) | 0.992 |  | -2.82 (-4.73, -0.91) | 0.004 |
| **≥60 years** |  |  |  |  |  |  |  |  |
| **Social isolation** |  |  |  |  |  |  |  |  |
| Cardiac index | 0.07 (-0.03, 0.17) | 0.161 |  | -0.06 (-0.15, 0.03) | 0.208 |  | 0.04 (-0.08, 0.16) | 0.522 |
| Cardiac output | 0.11 (-0.11, 0.33) | 0.326 |  | -0.09 (-0.27, 0.08) | 0.297 |  | 0.07 (-0.15, 0.30) | 0.524 |
| LVEF | -0.03 (-1.37, 1.30) | 0.962 |  | -0.51 (-1.93, 0.92) | 0.485 |  | 0.65 (-0.85, 2.16) | 0.397 |
| LVEDV | -3.19 (-9.33, 2.95) | 0.309 |  | -5.19 (-10.19, -0.19) | 0.042 |  | -3.41 (-10.06, 3.23) | 0.314 |
| LVESV | -0.63 (-4.46, 3.20) | 0.747 |  | -1.46 (-4.43, 1.51) | 0.336 |  | -2.24 (-6.06, 1.58) | 0.250 |
| LVSV | -1.46 (-5.34, 2.42) | 0.461 |  | -3.37 (-6.51, -0.24) | 0.035 |  | -0.83 (-4.43, 2.77) | 0.653 |
| **Loneliness** |  |  |  |  |  |  |  |  |
| Cardiac index | 0.03 (-0.08, 0.14) | 0.563 |  | 0.01 (-0.11, 0.13) | 0.861 |  | -0.07 (-0.16, 0.03) | 0.174 |
| Cardiac output | 0.05 (-0.15, 0.26) | 0.610 |  | -0.03 (-0.30, 0.24) | 0.835 |  | -0.07 (-0.24, 0.10) | 0.438 |
| LVEF | 0.58 (-0.78, 1.93) | 0.405 |  | 0.19 (-1.55, 1.93) | 0.834 |  | 0.73 (-0.55, 2.02) | 0.264 |
| LVEDV | 2.13 (-2.91, 7.18) | 0.407 |  | -4.19 (-10.67, 2.29) | 0.205 |  | -0.60 (-5.67, 4.46) | 0.816 |
| LVESV | 0.09 (-2.76, 2.94) | 0.949 |  | -1.29 (-5.44, 2.85) | 0.541 |  | -1.43 (-4.53, 1.67) | 0.367 |
| LVSV | 2.62 (-0.55, 5.79) | 0.105 |  | -2.65 (-6.96, 1.67) | 0.230 |  | 0.22 (-2.65, 3.10) | 0.878 |

^a^ *β* were adjusted for age, sex, ethnicity, current employment status, education level, Townsend deprivation index, smoking status, alcohol consumption frequency, physical activity, TV watching time, healthy diet score, and longstanding illnesses, disability, or infirmity.

CI=confidence interval; CMR=cardiac magnetic resonance; CVD=cardiovascular disease; LVEDV=left ventricular end diastolic volume; LVEF=left ventricular ejection fraction; LVESV=left ventricular end systolic volume; LVSV=left ventricular stroke volume.

The references here are participants who were never socially isolated or never lonely.

**Table S22. Associations of patterns of change in social isolation and loneliness with subsequent cardiac function by CMR stratified by age additionally adjusting marital status**

|  | **Transient** | |  | **Incident** | |  | **Persistent** | |
| --- | --- | --- | --- | --- | --- | --- | --- | --- |
|  | ***β* (95% CI) ^a^** | **P value** |  | ***β* (95% CI) ^a^** | **P value** |  | ***β* (95% CI) ^a^** | **P value** |
| **<60 years** |  |  |  |  |  |  |  |  |
| **Social isolation** |  |  |  |  |  |  |  |  |
| Cardiac index | -0.00 (-0.07, 0.07) | 0.966 |  | 0.04 (-0.02, 0.10) | 0.223 |  | 0.02 (-0.04, 0.08) | 0.468 |
| Cardiac output | -0.03 (-0.17, 0.11) | 0.640 |  | 0.02 (-0.10, 0.15) | 0.698 |  | 0.01 (-0.12, 0.14) | 0.902 |
| LVEF | 0.05 (-0.75, 0.86) | 0.898 |  | -0.18 (-0.89, 0.54) | 0.630 |  | -0.56 (-1.25, 0.14) | 0.118 |
| LVEDV | -1.77 (-5.50, 1.96) | 0.352 |  | -0.57 (-3.60, 2.46) | 0.713 |  | -1.84 (-5.64, 1.97) | 0.345 |
| LVESV | -0.40 (-2.47, 1.67) | 0.705 |  | 0.27 (-1.46, 2.00) | 0.761 |  | 0.38 (-1.72, 2.48) | 0.724 |
| LVSV | -1.42 (-3.55, 0.71) | 0.190 |  | -0.45 (-2.40, 1.51) | 0.654 |  | -1.98 (-4.16, 0.19) | 0.074 |
| **Loneliness** |  |  |  |  |  |  |  |  |
| Cardiac index | -0.05 (-0.11, 0.01) | 0.110 |  | 0.03 (-0.04, 0.09) | 0.388 |  | -0.09 (-0.15, -0.04) | 0.001 |
| Cardiac output | -0.11 (-0.23, 0.02) | 0.090 |  | 0.06 (-0.08, 0.20) | 0.407 |  | -0.17 (-0.28, -0.05) | 0.004 |
| LVEF | -0.03 (-0.67, 0.60) | 0.916 |  | 0.69 (-0.05, 1.43) | 0.070 |  | -0.57 (-1.23, 0.08) | 0.086 |
| LVEDV | -1.48 (-4.94, 1.99) | 0.403 |  | -1.32 (-5.13, 2.49) | 0.497 |  | -2.47 (-5.82, 0.87) | 0.147 |
| LVESV | -0.44 (-2.29, 1.40) | 0.637 |  | -1.13 (-3.26, 1.00) | 0.300 |  | -0.15 (-1.81, 1.52) | 0.864 |
| LVSV | -1.03 (-3.19, 1.13) | 0.352 |  | 0.11 (-1.97, 2.18) | 0.920 |  | -2.51 (-4.47, -0.55) | 0.012 |
| **≥60 years** |  |  |  |  |  |  |  |  |
| **Social isolation** |  |  |  |  |  |  |  |  |
| Cardiac index | 0.07 (-0.04, 0.17) | 0.229 |  | -0.07 (-0.16, 0.03) | 0.167 |  | 0.03 (-0.09, 0.15) | 0.640 |
| Cardiac output | 0.09 (-0.13, 0.31) | 0.406 |  | -0.11 (-0.29, 0.07) | 0.234 |  | 0.05 (-0.17, 0.28) | 0.649 |
| LVEF | -0.06 (-1.42, 1.31) | 0.937 |  | -0.52 (-1.94, 0.89) | 0.467 |  | 0.62 (-0.89, 2.13) | 0.422 |
| LVEDV | -3.48 (-9.70, 2.74) | 0.273 |  | -5.46 (-10.55, -0.37) | 0.036 |  | -3.82 (-10.48, 2.85) | 0.262 |
| LVESV | -0.73 (-4.66, 3.19) | 0.715 |  | -1.55 (-4.58, 1.48) | 0.317 |  | -2.38 (-6.27, 1.51) | 0.231 |
| LVSV | -1.66 (-5.65, 2.33) | 0.414 |  | -3.55 (-6.73, -0.36) | 0.029 |  | -1.13 (-4.77, 2.51) | 0.543 |
| **Loneliness** |  |  |  |  |  |  |  |  |
| Cardiac index | 0.03 (-0.08, 0.13) | 0.631 |  | 0.01 (-0.11, 0.13) | 0.898 |  | -0.08 (-0.17, 0.02) | 0.117 |
| Cardiac output | 0.04 (-0.16, 0.25) | 0.689 |  | -0.03 (-0.31, 0.24) | 0.821 |  | -0.09 (-0.26, 0.09) | 0.327 |
| LVEF | 0.57 (-0.78, 1.93) | 0.406 |  | 0.19 (-1.55, 1.93) | 0.834 |  | 0.73 (-0.56, 2.03) | 0.268 |
| LVEDV | 2.13 (-2.92, 7.19) | 0.409 |  | -4.19 (-10.67, 2.29) | 0.205 |  | -0.61 (-5.71, 4.50) | 0.816 |
| LVESV | 0.08 (-2.78, 2.94) | 0.956 |  | -1.30 (-5.45, 2.85) | 0.540 |  | -1.45 (-4.57, 1.67) | 0.363 |
| LVSV | 2.59 (-0.58, 5.76) | 0.109 |  | -2.65 (-6.96, 1.66) | 0.229 |  | 0.18 (-2.74, 3.09) | 0.905 |

^a^ *β* were adjusted for age, sex, ethnicity, current employment status, education level, Townsend deprivation index, smoking status, alcohol consumption frequency, physical activity, TV watching time, healthy diet score, and longstanding illnesses, disability, or infirmity.

CI=confidence interval; CMR=cardiac magnetic resonance; CVD=cardiovascular disease; LVEDV=left ventricular end diastolic volume; LVEF=left ventricular ejection fraction; LVESV=left ventricular end systolic volume; LVSV=left ventricular stroke volume.

The references here are participants who were never socially isolated or never lonely.

**Table S23. Associations of patterns of change in social isolation and loneliness with subsequent cardiac function by CMR stratified by age mutually adjusting social isolation score or loneliness score at baseline**

|  | **Transient** | |  | **Incident** | |  | **Persistent** | |
| --- | --- | --- | --- | --- | --- | --- | --- | --- |
|  | ***β* (95% CI) ^a^** | **P value** |  | ***β* (95% CI) ^a^** | **P value** |  | ***β* (95% CI) ^a^** | **P value** |
| **<60 years** |  |  |  |  |  |  |  |  |
| **Social isolation** |  |  |  |  |  |  |  |  |
| Cardiac index | -0.00 (-0.07, 0.07) | 0.928 |  | 0.03 (-0.03, 0.09) | 0.373 |  | 0.01 (-0.05, 0.08) | 0.654 |
| Cardiac output | -0.04 (-0.17, 0.10) | 0.614 |  | 0.01 (-0.11, 0.14) | 0.821 |  | -0.00 (-0.13, 0.13) | 0.990 |
| LVEF | 0.01 (-0.79, 0.81) | 0.986 |  | -0.25 (-0.96, 0.46) | 0.492 |  | -0.66 (-1.34, 0.02) | 0.057 |
| LVEDV | -1.81 (-5.54, 1.92) | 0.342 |  | -0.64 (-3.64, 2.36) | 0.677 |  | -1.94 (-5.73, 1.85) | 0.315 |
| LVESV | -0.37 (-2.45, 1.72) | 0.730 |  | 0.34 (-1.36, 2.05) | 0.692 |  | 0.50 (-1.56, 2.56) | 0.634 |
| LVSV | -1.53 (-3.64, 0.57) | 0.154 |  | -0.60 (-2.52, 1.33) | 0.543 |  | -2.23 (-4.40, -0.06) | 0.044 |
| **Loneliness** |  |  |  |  |  |  |  |  |
| Cardiac index | -0.06 (-0.12, 0.00) | 0.062 |  | 0.03 (-0.04, 0.09) | 0.419 |  | -0.10 (-0.15, -0.05) | <0.001 |
| Cardiac output | -0.12 (-0.24, 0.01) | 0.062 |  | 0.06 (-0.08, 0.20) | 0.383 |  | -0.17 (-0.28, -0.06) | 0.003 |
| LVEF | -0.10 (-0.73, 0.53) | 0.759 |  | 0.69 (-0.05, 1.44) | 0.068 |  | -0.62 (-1.27, 0.03) | 0.060 |
| LVEDV | -1.54 (-4.98, 1.90) | 0.380 |  | -1.33 (-5.13, 2.47) | 0.492 |  | -2.53 (-5.83, 0.77) | 0.133 |
| LVESV | -0.38 (-2.20, 1.45) | 0.684 |  | -1.14 (-3.25, 0.97) | 0.288 |  | -0.10 (-1.76, 1.55) | 0.905 |
| LVSV | -1.17 (-3.35, 1.02) | 0.294 |  | 0.13 (-1.94, 2.20) | 0.901 |  | -2.67 (-4.59, -0.74) | 0.007 |
| **≥60 years** |  |  |  |  |  |  |  |  |
| **Social isolation** |  |  |  |  |  |  |  |  |
| Cardiac index | 0.07 (-0.03, 0.18) | 0.161 |  | -0.06 (-0.15, 0.04) | 0.225 |  | 0.04 (-0.08, 0.16) | 0.493 |
| Cardiac output | 0.11 (-0.11, 0.33) | 0.315 |  | -0.09 (-0.27, 0.09) | 0.313 |  | 0.08 (-0.15, 0.30) | 0.501 |
| LVEF | -0.05 (-1.41, 1.31) | 0.945 |  | -0.53 (-1.95, 0.88) | 0.459 |  | 0.63 (-0.87, 2.13) | 0.413 |
| LVEDV | -3.23 (-9.45, 3.00) | 0.309 |  | -5.27 (-10.29, -0.25) | 0.040 |  | -3.50 (-10.13, 3.13) | 0.301 |
| LVESV | -0.62 (-4.45, 3.21) | 0.751 |  | -1.44 (-4.42, 1.54) | 0.343 |  | -2.21 (-6.05, 1.62) | 0.257 |
| LVSV | -1.55 (-5.56, 2.47) | 0.450 |  | -3.51 (-6.73, -0.28) | 0.033 |  | -0.95 (-4.54, 2.64) | 0.603 |
| **Loneliness** |  |  |  |  |  |  |  |  |
| Cardiac index | 0.03 (-0.08, 0.14) | 0.588 |  | 0.01 (-0.11, 0.13) | 0.882 |  | -0.07 (-0.17, 0.02) | 0.143 |
| Cardiac output | 0.05 (-0.15, 0.26) | 0.621 |  | -0.03 (-0.30, 0.25) | 0.834 |  | -0.07 (-0.24, 0.10) | 0.423 |
| LVEF | 0.57 (-0.79, 1.92) | 0.411 |  | 0.18 (-1.56, 1.92) | 0.839 |  | 0.71 (-0.58, 2.00) | 0.278 |
| LVEDV | 2.34 (-2.76, 7.44) | 0.368 |  | -3.98 (-10.51, 2.54) | 0.232 |  | -0.23 (-5.18, 4.73) | 0.929 |
| LVESV | 0.19 (-2.66, 3.05) | 0.894 |  | -1.24 (-5.50, 3.02) | 0.569 |  | -1.25 (-4.31, 1.81) | 0.424 |
| LVSV | 2.74 (-0.44, 5.93) | 0.091 |  | -2.59 (-6.92, 1.74) | 0.241 |  | 0.39 (-2.47, 3.24) | 0.791 |

^a^ *β* were adjusted for age, sex, ethnicity, current employment status, education level, Townsend deprivation index, smoking status, alcohol consumption frequency, physical activity, TV watching time, healthy diet score, longstanding illnesses, disability, or infirmity, and social isolation or loneliness. The references here are participants who were never socially isolated or never lonely.

CI=confidence interval; CMR=cardiac magnetic resonance; CVD=cardiovascular disease; LVEDV=left ventricular end diastolic volume; LVEF=left ventricular ejection fraction; LVESV=left ventricular end systolic volume; LVSV=left ventricular stroke volume.
